# Pre-pregnancy complications - associated factors and wellbeing in early pregnancy: a Swedish cohort study

**DOI:** 10.1186/s12884-023-05479-8

**Published:** 2023-03-08

**Authors:** Unnur Gudnadottir, Juan Du, Luisa W. Hugerth, Lars Engstrand, Ina Schuppe-Koistinen, Eva Wiberg Itzel, Emma Fransson, Nele Brusselaers

**Affiliations:** 1 Centre for Translational Microbiome Research, Department of Microbiology, Tumor and Cell Biology (MTC), Karolinska Institutet, 171 65 Solna, Sweden; 2 Department of Medical Biochemistry and Microbiology, Uppsala University, Uppsala, Sweden; 3 Science for Life Laboratory, 171 65, Solna, Sweden; 4 Department of Clinical Science and Education, Södersjukhuset, Stockholm, Sweden; 5 Department of Women’s and Children’s health, Uppsala University, 751 85 Uppsala, Sweden; 6 Global Health Institute, University of Antwerp, 2610 Antwerp, Belgium; 7 Department of Head and Skin, Ghent University, 9000 Ghent, Belgium

**Keywords:** Artificial reproductive technologies, Early pregnancy, Fertility, Recurrent pregnancy loss, Subfertility, Late miscarriage, Pregnancy symptoms, Pre-pregnancy

## Abstract

**Background:**

Many couples experience difficulties to become pregnant or carry a pregnancy to term due to unknown causes. Here we define pre-pregnancy complications as having prior recurrent pregnancy loss, prior late miscarriages, time to pregnancy more than one year, or the use of artificial reproductive technologies. We aim to identify factors associated with pre-pregnancy complications and poor well-being in early pregnancy.

**Methods:**

Online questionnaire data from 5330 unique pregnancies in Sweden were collected from November 2017 – February 2021. Multivariable logistic regression modelling was used to investigate potential risk factors for pre-pregnancy complications and differences in early pregnancy symptoms.

**Results:**

Pre-pregnancy complications were identified in 1142 participants (21%). Risk factors included diagnosed endometriosis, thyroid medication, opioids and other strong pain medication, body mass index > 25 kg/m^2^ and age over 35 years. Different subgroups of pre-pregnancy complications had unique risk factors. The groups also experienced different pregnancy symptoms in early pregnancy, where women that had experienced recurrent pregnancy loss were at higher risk of depression in their current pregnancy.

**Conclusion:**

We report one of the largest pregnancy cohorts with high frequency of pre-pregnancy complications compared to the Swedish population. Prescribed drug use and body weight were the top potentially modifiable risk factors in all groups. Participants that experienced pre-pregnancy complications also had higher risk of depression and pregnancy problems in early pregnancy.

**Supplementary Information:**

The online version contains supplementary material available at 10.1186/s12884-023-05479-8.

## Background

There are many unanswered questions regarding a pre-pregnancy complications and early pregnancy well-being. Pre-pregnancy complications could be due to repeated miscarriages (recurrent pregnancy loss, RPL), having experienced a late miscarriage, prolonged time to conception (subfertility) or needing assisted reproductive technologies (ART). Many couples experience difficulties to become pregnant or carry a pregnancy to term due to unknown causes.

Approximately 2.5% of women worldwide suffer from RPL [1], defined as ≥ 2 (United States) or ≥ 3 (United Kingdom) consecutive miscarriages [2]. Common causes are genetic, uterine abnormalities, endocrine disorders and obesity, but half of cases have unknown causes [2].

Subfertility is defined as failure to conceive within one year of unprotected intercourse – a delay or reduction in fertility [3]. A woman’s age is a major influence on fertility, which starts decreasing from 25 to 30 years of age [3, 4]. Overall, subfertility and infertility are estimated to affect 10–15% of couples [5]. It has been reported that pre-existent obesity [6], smoking, or excessive caffeine or alcohol consumption [4] can reduce fertility. Other causes include polycystic ovary syndrome (PCOS), endometriosis, damage to the fallopian tubes (e.g., by *Chlamydia trachomatis* infection*)* and male subfertility [3–5, 7]. However, many couples have unexplained subfertility [4, 5, 7]. It is estimated that 84% of couples conceive within one year of trying [4, 7], which is often used to define subfertility [8] and a common benchmark before starting ART [4].

ART include in vitro fertilization (IVF), intrauterine insemination (IUI) and intracytoplasmic sperm injection (ICSI) [4]. In Sweden, the use of IVF and ISCI has increased steadily over the last decades [9], with approximately 4.5% of children in Sweden are conceived by IVF every year [10].

Most current studies on reproductive history and pregnancy focus on birth outcome and not potential risk factors for the difficulties conceiving or maintaining the pregnancy. Fertility complications can cause increased anxiety, stress and self-blame [11], which in turn can negatively affect fertility [4, 12]. If pre-pregnancy complications affect the early phase of pregnancy and pregnancy symptoms also remains unclear. In this study, we aim to identify potential risk factors for pre-pregnancy complications, as well as assessing wellbeing in early pregnancy.

## Methods

### Aim

Here we use questionnaire data from the Swedish Maternal Microbiome (SweMaMi) cohort study [13] to identify potential risk factors for pre-pregnancy complications in comparison to having none.

Our second aim is to assess the differences in pre-pregnancy complications subgroups: RPL, subfertility, ART and late miscarriage, compared to the uncomplicated pre-pregnancy group and differences in early pregnancy symptoms.

### Study design- and period

The SweMaMi project started enrolment in November 2017 and ended February 2021 (with last deliveries during autumn 2021). All pregnant women (before gestational week 20) residing in Sweden with a personal identification number were eligible to participate. Since no additional healthcare visits were required to participate in the study, women all over Sweden could participate [13].

The study was performed according to the Declaration of Helsinki and approved by the regional Ethics Committee of Stockholm (Dnr 2020 − 01629). The study cohort is described in detail in a cohort profile [13]. In short, participants gave their informed consent when completing the first online questionnaire. We collected data at three different timepoints, starting with an online questionnaire, followed by home-sampling for microbiome assessment: (i) Before pregnancy week 20, (ii) Pregnancy week 28–30 and (iii) 5–8 weeks postpartum.

In addition, a registry linkage will be conducted to obtain more detailed information from the Medical Birth Registry, Patient Registry and Drug Registry.

### Data collection and eligibility

The three questionnaires included questions regarding pregnancy, lifestyle, physical-, mental- and reproductive health. The last questionnaire focused more on the birth itself and the infant’s health. For the present study, only the first questionnaire (baseline) is used (Supplement 1). Single women were excluded because of the potential social reasons for pre-pregnancy complications. Participants that did not answer their number of previous early miscarriages or previous late miscarriages (including intrauterine fetal demise (IUFD)) were considered as having had none. Other missing answers were regarded as missing and omitted from the multivariable analyses.

### Pre-pregnancy complications

Pre-pregnancy complications were defined based on a history of (i) previous three or more early miscarriages [2], (ii) previous late miscarriage (after week 16) including IUFD, or for the current pregnancy (iii) ART (IVF, insemination, or other medically or surgical assistance), or (iv) duration exceeding one year to conceive.

Uncomplicated pre-pregnancy was defined as pregnancies with fewer than three prior early miscarriages, no late miscarriage or IUFD, less than one year to conceive and unassisted conception. If a participant did not answer a question needed for this categorization, the answer was considered uncomplicated.

Questions regarding potential risk factors were chosen from the questionnaire to estimate: (i) general health and health seeking behavior (body mass index (BMI), menstruation, cervical screening attendance, eating disorders and gynecological infections), (ii) lifestyle (age, country of birth, education level, work situation, contact with animals, smoking, mouth tobacco use and diet), (iii) drugs (drug use before pregnancy: asthma and allergy medication, anxiety, antidepressants and sleep medication, prescription free pain medication, opioids and strong pain medication, thyroid medication, blood pressure medication, stomach acid medication and other medication), (iv) reproductive health (first pregnancy and contraceptive use) and (v) comorbidities (diagnosed and suspected endometriosis and polycystic ovary syndrome (PCOS)) (Supplement 1). Participants were sorted as having suspected PCOS if they reported Ferriman-Gallwey score ≥ 8 [14] or hair loss grade 3, and suspected endometriosis if they reported vaginal spotting before the start of menstruation [15, 16].

### General health problems

To estimate the effect of general health prior to pregnancy on the risk of pre-pregnancy complications, six categories were created based on reported medical history: (i) Gynecological history (diagnosed or suspected endometriosis or PCOS), (ii) Non-gastrointestinal chronic inflammatory diseases (diagnosed rheumatoid arthritis, autoimmune diseases or multiple sclerosis (MS)), (iii) Gastrointestinal problems (gastric acid medication, diagnosed Crohn’s disease or ulcerative colitis), (iv) Mental health (eating disorder, anti-depressive-, anti-anxiety- or sleeping medication), (v) Chronic respiratory diseases and allergies (asthma, hay fever, allergies, asthma- or allergy medication) and (vi) Endocrine indications (thyroid medication or diagnosed hypothyroidism). These categories were not mutually exclusive. Participants with each comorbidity were compared to those without the specific comorbidity; and the group with any of these comorbidities was also compared to those without.

### Early pregnancy well-being and characteristics

To assess if early pregnancy well-being and characteristics are different among participants with pre-pregnancy complications compared to those without, data regarding depressive symptoms (Edinburgh Postnatal Depression Scale [17]), stress (Cohen Perceived Stress Scale [18]), bowel symptoms (Bristol stool form scale [19]), pregnancy problems, vomiting and nausea [20], self-reported general health estimate, alcohol consumption [21] and medication (drug use during pregnancy: asthma and allergy medication, anxiety, antidepressants and sleep medication, prescription free pain medication, opioids and strong pain medication, thyroid medication, blood pressure medication, stomach acid medication and other medication) were extracted from the questionnaire.

### Statistical analyses

Statistical analyses were conducted in Excel (version 2108) and RStudio (version 1.3.1093). Descriptive statistics are presented as absolute numbers, percentages and means with standard deviation.

Multivariable logistic regression modelling was used to investigate potential risk factors as exposure with pre-pregnancy complications (and its subgroups) as outcome, using the total group with an uncomplicated pre-pregnancy onset as reference for all analyses. The same method was used for the different categories of health problems.

Multivariable logistic regression was then performed to investigate the odds of early pregnancy symptoms as outcome, in participants with pre-pregnancy complications as exposure.

All results (independent of statistical significance) are presented as odds ratios (OR) and 95% confidence intervals.

## Results

**Fig. 1:**
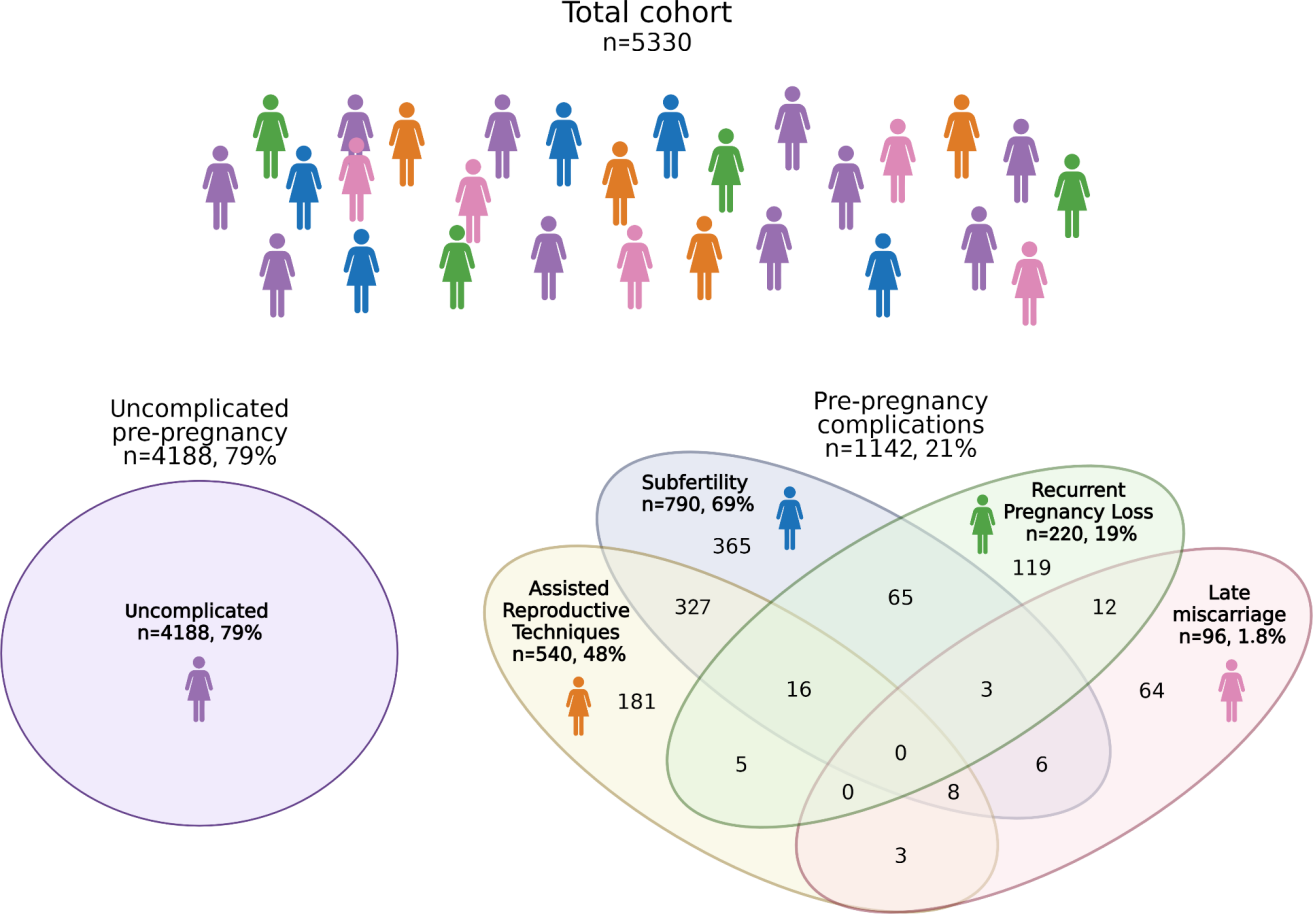
Categorization of the cohort as uncomplicated pre-pregnancy and pre-pregnancy complications with subgroups. Generated with BioRender

Out of the total 5330 eligible pregnancies (5144 unique participants), 1142 (21%) were categorized as having pre-pregnancy complications (Table 1). Out of the pre-pregnancy complications, 220 (19%) participants had suffered RPL, 790 (69%) subfertility, 96 (8%) had a previous late miscarriage and 540 (47%) used ART (Fig. 1). The largest overlap comprised subfertile participants who got pregnant with the help of ART (n = 327, 29%), and subfertile participants who experienced RPL (n = 65, 6%) (Fig. 1). The mean time to get pregnant was 19 months for the pre-pregnancy complications group and 2 months for the uncomplicated group.

**Table 1:** Definitions of pre-pregnancy complications and uncomplicated pre-pregnancy

		Total cohort (n = 5330)		Uncomplicated (n = 4188)		Pre-pregnancy complications (n = 1142)	
		n	%	n	%	n	%
**Number of previous early miscarriages**	0	3672	68.9	3100	74.0	572	50.1
	1–2	1438	27.0	1088	26.0	350	30.6
	≥ 3	220	4.1	0	0.0	220	19.3
**Previous late miscarriages (after week 16) or IUFD**	Yes	96	1.8	0	0.0	96	8.4
	No	5234	98.2	4188	100.0	1046	91.6
**Method of conception**	Uncomplicated	4850	91.0	4187	100.0	663	58.1
	In vitro fertilization (IVF)	307	5.8	2	0.0	305	26.7
	Intracytoplasmic sperm injection (ICSI)	98	1.8	2	0.0	96	8.4
	Insemination	53	1.0	3	0.1	50	4.4
	Medically or surgically assisted	138	2.6	23	0.5	100	8.8
**Time to pregnancy**	< 6 months	3835	72.0	3601	86.0	234	20.5
	6–12 months	642	12.0	536	12.8	106	9.3
	>= 12 months	790	14.8	0	0.0	790	69.2
	Missing	63	1.2	51	1.2	12	1.1

IUFD, Intrauterine fetal demise

The average gestational length when answering the questionnaire was similar in all four subgroups of pre-pregnancy complications and uncomplicated pre-pregnancy (12 weeks), allowing comparison for pregnancy related questions, such as pregnancy problems and symptoms.

### Characteristics of pre-pregnancy complications

**Fig. 2:**
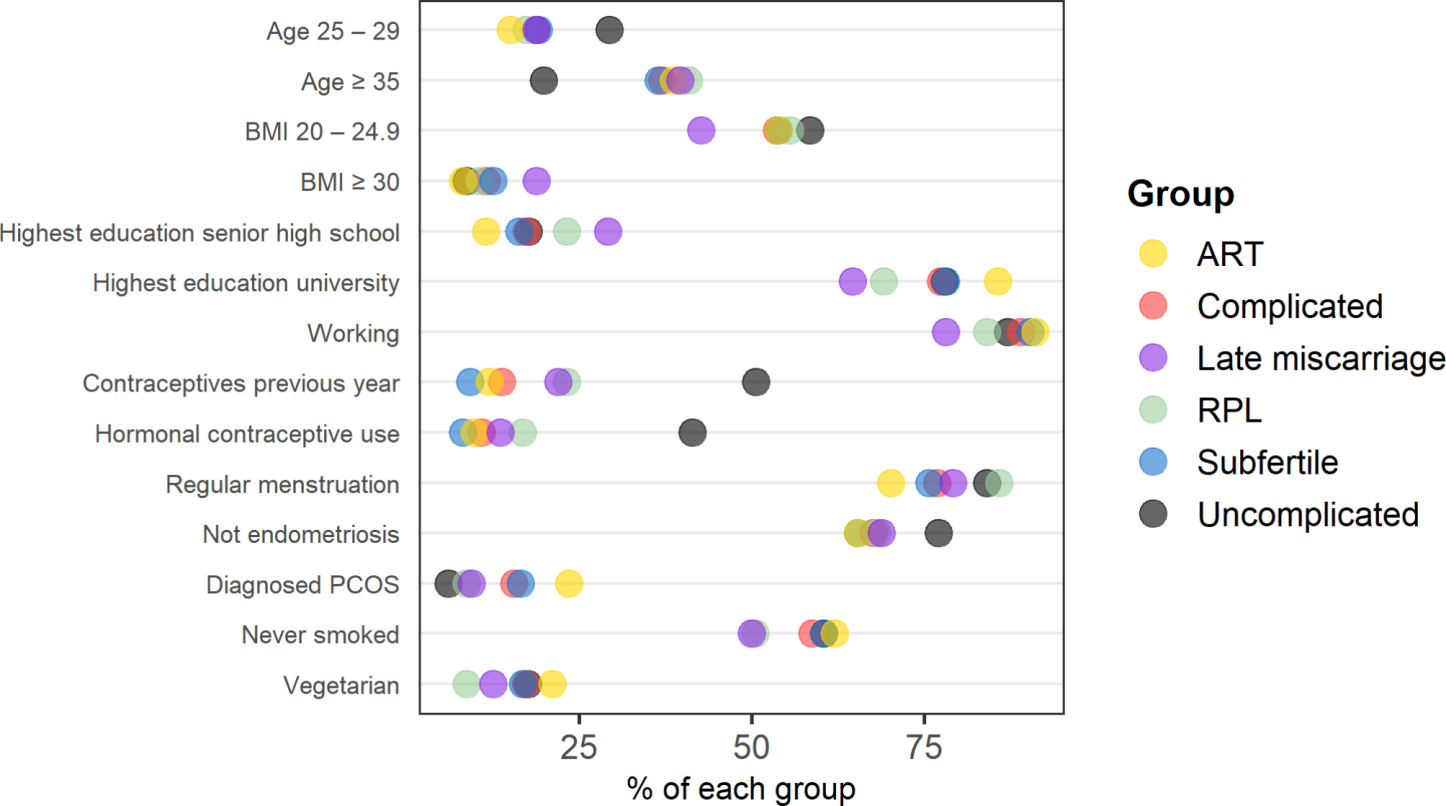
Descriptive statistics of the uncomplicated pre-pregnancy and pre-pregnancy complications group, and its subgroups, where the difference between the highest and lowest subgroup value was over 10%. Complete dataset in Supplementary Table 3

The pre-pregnancy complications group was more likely to be overweight (BMI > 25 kg/m^2^) or obese (BMI > 30 kg/m^2^) (35% vs. 28%) and older than 35 years (37% vs. 20%) than the uncomplicated pre-pregnancy group. Country of birth, education level and working situation were similar for both groups (Supplementary Table 1). Characteristics with between-group differences larger than over 10% were listed in Fig. 2.

### Characteristics of the subgroups of pre-pregnancy complications

Descriptive analyses showed that out of the four complicated subgroups, participants who experienced RPL had the highest prevalence of using asthma or allergy medication (21% vs. 15% uncomplicated) and the lowest representation of vegetarians (9% vs. 18% uncomplicated) (Supplementary Tables 1, Fig. 2). Subfertile participants were the youngest of the complication’s subgroups (22% under 30 years old vs. 33% uncomplicated). Out of the four subgroups, participants becoming pregnant via ART had the highest prevalence of completed university education (86% vs. 78% uncomplicated), having diagnosed endometriosis (12% vs. 2% uncomplicated) and PCOS (24% vs. 6%) and reported irregular menstruation prior to this pregnancy (29% vs. 16% uncomplicated).

### Factors associated with pre-pregnancy complications

The following factors were identified as being associated with pre-pregnancy complications compared to uncomplicated: age ≥ 35 years (OR 2.84, CI 1.90–4.40), being overweight or obese (OR 1.35, CI 1.07–1.72 for overweight and OR 1.58, CI 1.19–2.07 for obese), born outside Sweden (OR 1.42, CI 1.15-1-76), irregular menstruation (OR 1.59, CI 1.35–1.87), diagnosed (OR 4.43, CI 3.33–5.91) and suspected (OR 1.29, CI 1.09–1.51) endometriosis, diagnosed PCOS (OR 2.89, CI 2.35–3.54), regular contact with animals (OR 1.17, CI 1.03–1.34), and the use of certain drugs prior to pregnancy (asthma and allergy medication (OR 1.23, CI 1.03–1.46), opioids and strong pain medication (OR 1.97, CI 1.19–3.19) and thyroid medication (OR 2.20, CI 1.76–2.74) (Supplementary Table 2).

**Fig. 3:**
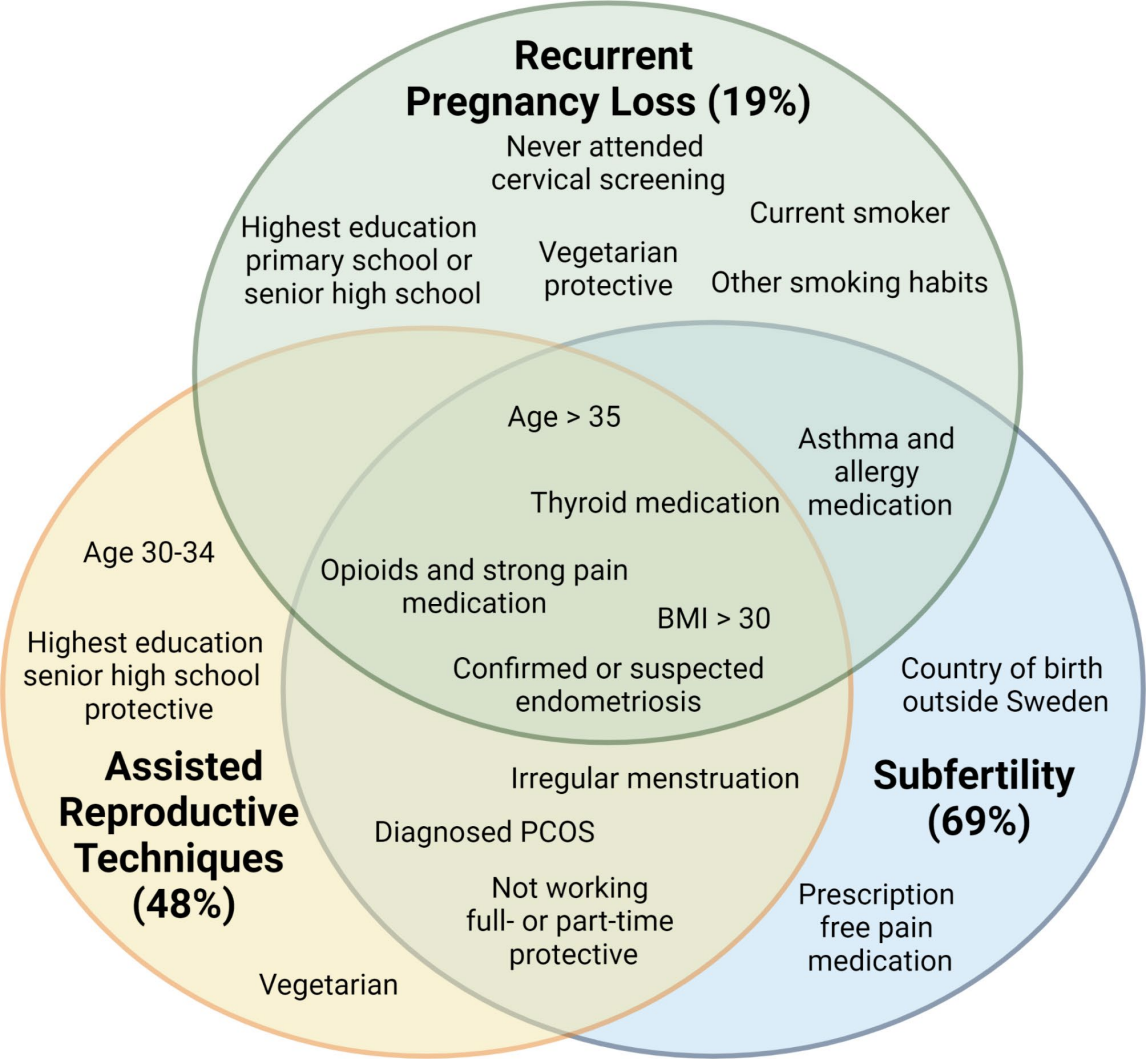
Venn diagram of risk factors for the different categories of pre-pregnancy complications, shown with percentage of complicated cases. Overlapping circles show risk factors for multiple groups. Generated with BioRender

Logistic regression for RPL, subfertility and ART showed that they had different and sometimes unique risk factors (Supplementary Tables 2, Fig. 3). The late miscarriage subgroup was not included because of the small group size (n = 96).

Unique risk factors for the RPL group were not having attended cervical screening (OR 1.69, CI 1.00-2.69), being a current smoker (OR 4.39, CI 2.06–8.51), and lower education levels (OR 3.64, CI 1.65–7.17 for primary school as highest education level and OR 1.49, CI 1.06–2.05 for senior high school as highest education level). Furthermore, being vegetarian appeared protective for the RPL group (OR 0.45, CI 0.27–0.70).

Unique risk factors for subfertility were country of birth outside Sweden (OR 1.58, CI 1.24-2.00) and use of prescription-free pain medication prior to pregnancy (OR 1.22, CI 1.03–1.44).

Moreover, the unique risk factors for ART were age 30–34 years (OR 2.77, CI 1.38–5.59), and being vegetarian (OR 1.27, CI 1.01–1.58), and senior high school education level protective (OR 0.59, CI 0.45–0.78).

### General health and the risk of pre-pregnancy complications

Out of the total cohort (n = 5330), 65% belonged to at least one category of health estimation – indicating that only one third of the cohort could be regarded as entirely “healthy” according to our categorization (Supplementary Table 3). There were higher odds for pre-pregnancy complications in all the six subgroups, except for chronic inflammatory diseases which showed no association (OR 0.98, CI 0.62–1.51).

**Fig. 4:**
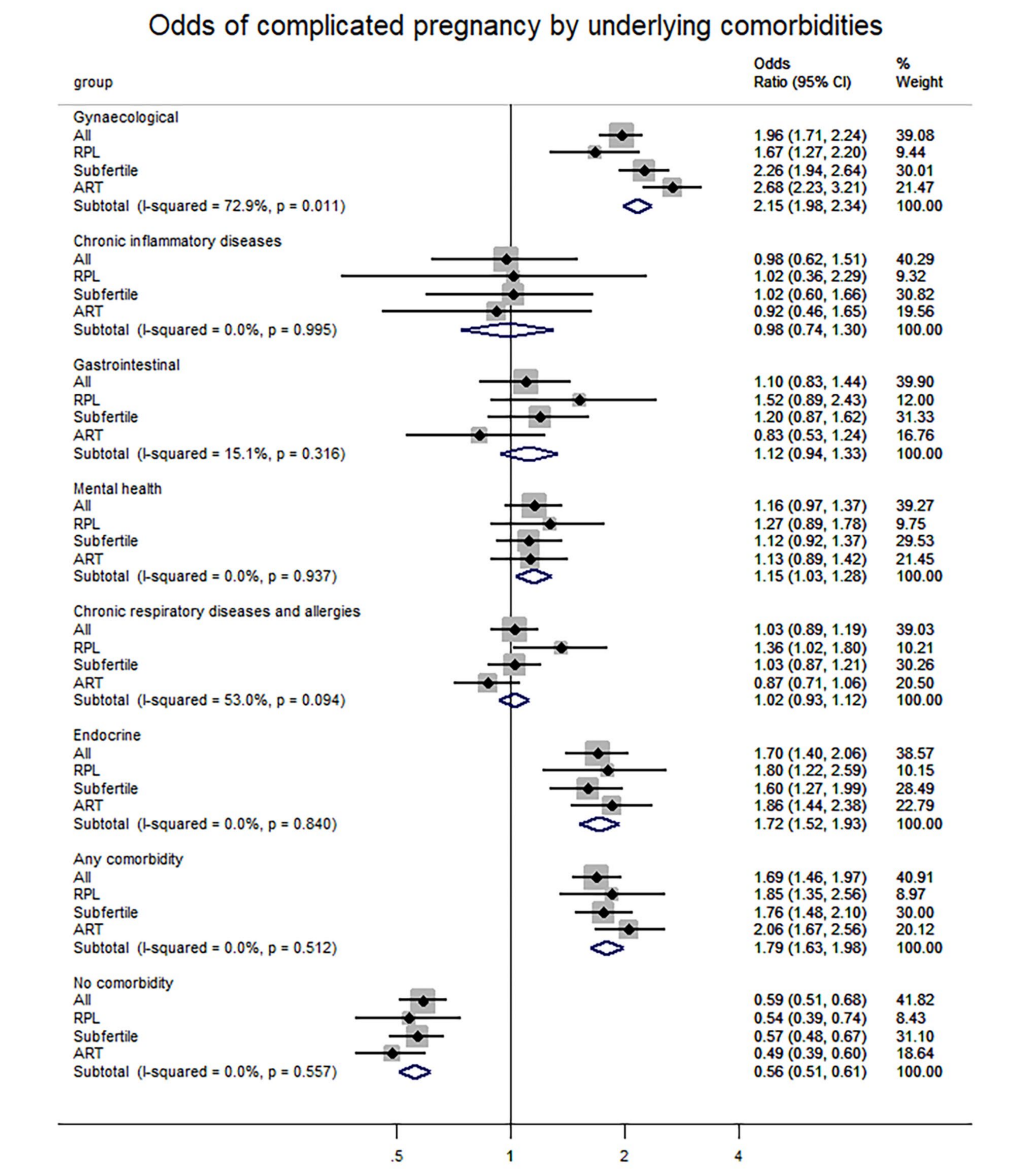
Forest plot showing odds of a pre-pregnancy complications by underlying comorbidities, with 95% confidence intervals. Not having the specific comorbidity was used as reference. Power of each analysis is indicated by the size of the gray boxes

Having gynecological (OR 1.94, CI 1.71–2.24), endocrine comorbidities (OR 1.70, CI 1.40–2.06) or belonging to any of the six chronic comorbidities categories (OR 1.69, CI 1.46–1.97) increased the risk of pre-pregnancy complications (Fig. 4). For participants that had chronic respiratory diseases and allergies (OR 1.36, CI 1.02–1.80), the risk of RPL was also significantly higher (Supplementary Table 3). The late miscarriage group was excluded from individual analysis because of sample size.

### Early pregnancy well-being for pre-pregnancy complications subgroups

Compared to uncomplicated pre-pregnancy, participants who had pre-pregnancy complications were more likely to have experienced pregnancy-related problems (17% vs. 13% uncomplicated), where depression (3% vs. 1% uncomplicated), vaginal bleeding (4% vs. 2% uncomplicated) and acid reflux (6% vs. 4% uncomplicated) were the most common problems observed in our cohort (Supplementary Table 4).

Similar percentage of participants in both the complicated and uncomplicated pre-pregnancy groups considered their health as either very good or pretty good (90% vs. 92% uncomplicated). Participants that had suffered RPL or a late miscarriage had the highest perceived stress (mean 5.77 and 5.86 respectively vs. 5.43 for uncomplicated) and scored highest on the EPDS (mean 8.17 and 8.71 respectively vs. 7.02 for uncomplicated). Out of all four subgroups, participants in the late miscarriage group had highest prevalence of nausea for more than 6 h daily (20% vs. 15% uncomplicated).

### Risk factors for experiencing physical and psychological symptoms

Out of the subgroups, participants that had experienced RPL showed a significantly higher risk of depression (OR 1.71, CI 1.21–2.38) and constipation (OR 1.36, CI 1.02–1.80) (Supplementary Table 4).

All groups showed higher risk of pregnancy problems and taking opioids and strong pain medication, thyroid medication and other medication during pregnancy (Supplementary Table 4).

## Discussion

Besides the known risk factors age and overweight/obesity [2–4, 6], prescribed drug use stood out as a factor associated with being in almost all groups with pre-pregnancy complications, yet we cannot distinguish between the effect of the drugs and confounding by indication. The RPL group seemed to have the most socio-economic risk factors, including low education, smoking and having never attended cervical screening, which could indicate more limited healthcare-seeking behavior. The ART and subfertility groups were largely overlapping considering risk factors, as were the subfertility group and overall pre-pregnancy complications. For subfertility and ART, higher education level, having a job, no use of contraceptives and higher age could be risk factors since the participants have likely attempted pregnancy for a long time before being categorized as subfertile or qualifying for ART.

Our results also point to a difference in early pregnancy well-being, where participants with pre-pregnancy complications tend to have higher levels of anxiety and depression, are more likely to have experienced any pregnancy complications and have used drugs during pregnancy than the uncomplicated group.

Very little research has been conducted on pre-pregnancy complications. Endometriosis, PCOS and age have previously been described as risk factors for subfertility [3–5, 7]. In line with earlier studies, we also found that endocrine and gynecological comorbidities had the biggest effect on the risk of pre-pregnancy complications. Moreover, with our extensive questionnaires, we were also able to identify certain prescribed drug groups and country of birth (thereby, being an immigrant) as possible risk factors.

Modifiable factors such as weight loss, smoking cessation, and only taking drugs when truly needed (and doing so with proper guidance) could potentially increase the chances of an uncomplicated pre-pregnancy. Our results also suggest that the microbiome might have a role in the risk of RPL, where drugs had the highest effect [22, 23]. However, potential confounding by indication needs to be addressed.

Currently there is no standardized approach for RPL in Sweden, due to many cases have unknown causes and the molecular mechanisms of RPL are greatly understudied [24]. Knowledge of the identified risk factors might assist the healthcare system to identify women at higher risk of subfertility and/or RPL and follow them up more closely with support and counsel when trying to conceive. Individuals at risk might be aided through nutritional advise to help weight loss, overview of medicinal intake or psychotherapy to minimize the risk of stress and depression. The risk factors found in our study could also be used to increase fertility awareness. Further studies are needed to better understand the effect of certain drug groups and the underlying mechanisms of their risks for each subgroup of pre-pregnancy complications.

We constructed one of the largest pregnancy cohorts enrolling women from all over Sweden with detailed, standardized and validated questions. With over 5300 women in our cohort we represent approximately 1.5% of all pregnancies in Sweden during the study period (estimated 115–120,000 pregnancies each year [10]). We have a high number of participants from the Stockholm region (c. 40% of total cohort) and high education level, which can be expected in scientific studies with population-wide enrollment. Furthermore, participants born outside of Sweden are underrepresented, possibly because of language barriers. However, in our recent cohort profile [13], we found that our cohort was fairly representative of the Swedish pregnant population.

To our knowledge, this is the first large study to estimate the risk factors for pre-pregnancy complications, and how pre-pregnancy complications displays in early pregnancy.

There are also limitations of this study. Some participants did not answer all the questions, for example number of previous early miscarriages (n = 1572, 30% of cohort), which were interpreted as having had none. Furthermore, we do not have information on the partner’s medical history, so we cannot exclude the subfertility and the need for ART might be due to the partner’s condition. We also have a high prevalence of participants with RPL, 4.1% (compared to estimated 2.5% worldwide [1]), and IVF, 5.8% (compared to 4.5% in Sweden yearly [10]), which might indicate a slight selection bias towards participants with complicated pregnancy onset. Because of their past experiences, RPL participants might be more eager to participate and contribute to research in this field. It could also point to an underestimation of RPL in the literature, since most miscarriages occur before 12 completed weeks of pregnancy, before many women are registered pregnant in the healthcare system, and therefore are not counted in the population statistics. The same holds true for a high number of women with suspected PCOS and endometriosis in the cohort, two gynecological comorbidities which are vastly underdiagnosed in the general population [25, 26], but have a great impact on pregnancy onset.

Further research on women’s health and pre-pregnancy complications is of high importance, since so many women and couples suffer involuntary childlessness, problems to get pregnant or stay pregnant, with no answers to the underlying cause.

## Conclusion

We reported one of the largest pregnancy cohorts with high frequency of pre-pregnancy complications (21%). We evaluated and presented different risk profiles for four pre-pregnancy complications subgroups: RPL, subfertility, ART and late miscarriage. Prescribed drug use and body weight are the major potentially modifiable risk factors in all groups. Furthermore, women with a pre-pregnancy complications had a higher risk of pregnancy complications in their current pregnancy. The identified risk factors could be used to identify women at risk for pre-pregnancy complications and help them earlier in the process of trying to conceive, thus assisting them to reduce stress and depression during the pregnancy.

## Electronic supplementary material

Below is the link to the electronic supplementary material.

**Supplementary material 1**

**Supplementary material 2**

## Data Availability

The datasets analyzed during the current study are not publicly available because of the high detail of clinical and personal information (despite being depersonified) - but are available from the corresponding author on reasonable request.

## Abbreviations

ART: Assisted reproductive technologies
BMI: Body mass index
CI: Confidence interval
ICSI: Intracytoplasmic sperm injection
IUFD: Intrauterine fetal demise
IUI: Intrauterine insemination
IVF: In vitro fertilization
MS: Multiple sclerosis
OR: Odds ratio
PCOS: Polycystic ovary syndrome
RPL: Recurrent pregnancy loss
