# Supplementary material for: Pre-pregnancy complications - associated factors and wellbeing in early pregnancy: a Swedish cohort study
Source: BMC Pregnancy Childbirth. 2023 Mar 8;23:153. doi: 10.1186/s12884-023-05479-8 (PMC9993650; doi:10.1186/s12884-023-05479-8)
Supplement: Supplementary file 1 — Supplementary material 1 [file 12884_2023_5479_MOESM1_ESM.docx]

**Supplementary material**

**Data extracted from baseline questionnaire and its categorization**

| **Question** | **Answers from questionnaire** | **Categorization** |
| --- | --- | --- |
| **Factors before pregnancy** | | |
| Family status | Single or Not |  |
| Work situation | Other, Working part-time, Working full-time, Unemployed, Long-term illness, Student. | Working (part-time and full-time) and Other (all other groups) |
| Highest level of education | Primary school, Senior high school, University / college, Other |  |
| Prior children | Yes or No |  |
| Age | Continuous numeric | < 25, 25 – 29, 30 – 34, ≥ 35 |
| BMI before pregnancy | Continuous numeric | < 20, 20 – 24.9, 25 – 29.9, ≥ 30 |
| Contraceptive use before pregnancy | Yes or No |  |
| Type of contraceptive used | The pill, Progestogen-only injectable contraceptive, Hormonal implant, Vaginal ring, Copper intrauterine device, Hormonal intrauterine device, Condoms, Diaphragm, Natural family planning, Other | Hormonal (The pill, Progestogen-only injectable contraceptive, Hormonal implant, Vaginal ring, Copper intrauterine device, Hormonal intrauterine device) or Non-hormonal (Condoms, Diaphragm, Natural family planning, Other) |
| Regular menstruation | Yes (ca 28 days), No (≥35 days), No (less than 3/year), No (less than 6/year) | Yes or No |
| PCOS | Yes, No, Don’t know  Hair loss grade 1, 2 and 3  Ferriman-Gallway score 0-36 | Yes, Maybe (hair loss grade 3 or Ferriman-Gallway > 8) or No |
| Endometriosis | Yes (diagnosed after operation), Yes (diagnosed without operation), No and Don’t know  Spotting before menstruation starts (Yes or No) | Yes, Maybe (Yes to spotting before menstruation starts) or No |
| Gynecological infections | Chlamydia, Gonorrhea, Bacterial vaginosis, Genital herpes, *Mycoplasma genitalium*, Fungal infection, Condyloma, *Trichomonas vaginalis*, Other | Sexually transmitted diseases (STDs) (Chlamydia, Gonorrhea, Genital herpes, *Trichomonas vaginali*s), Bacterial vaginosis, Fungal infection, *Mycoplasma genitalium*, Condyloma, Other and Any infection |
| Eating disorder | Yes or No | Yes or No |
| Regular contact with animals | Yes or No | Yes or No |
| Ever attended cervical screenings | Yes or No | Yes or No |
| Ever had cell changes | Yes or No | Yes or No |
| Smoking | Yes, Smoked previously, Smoke at parties, Never smoked, Other | Never, Current smokers (Yes) and Other (Smoked previously, Smoke at parties and Other) |
| Mouth tobacco | Yes, Used snuff previously, Other, Never used snuff | Never, Current users, Other (Other, used snuff previously) |
| Vegetarian | Yes, Yes, but not during pregnancy, No | Yes or No |
| Eats fish | Yes or No | Yes or No |
| Sweet drinks | Daily, often per week, once a week, seldom | Often (daily, often per week) or Seldom (once a week, seldom) |
| Sugarfree drinks | Daily, often per week, once a week, seldom | Often (daily, often per week) or Seldom (once a week, seldom) |
| Wholegrain bread | Daily, often per week, once a week, seldom | Often (daily, often per week) or Seldom (once a week, seldom) |
| Fruit | Daily, often per week, once a week, seldom | Often (daily, often per week) or Seldom (once a week, seldom) |
| Vegetables | Daily, often per week, once a week, seldom | Often (daily, often per week) or Seldom (once a week, seldom) |
| Drug use before pregnancy | Prescription free pain medication, opioids/strong pain medication, allergy medication, asthma medication, antidepressant medication, anxiety medication, sleeping pills, thyroid hormones, blood pressure medication, stomach acid medication or other | (1) Asthma and allergy medication, (2) Anxiety, antidepressants and sleep medication, (3) Prescription free pain medication, (4) Opioids and strong pain medication, (5) Thyroid medication, (6) Blood pressure medication, (7) Stomach acid medication, (8) Other |
| **Factors related to pregnancy** | | |
| Pregnancy complications | Yes or No  Pregnancy diabetes, Hyperthyroidism, Hypothyroidism, High blood pressure, Preeclampsia, Hyperemesis gravidarum, Depression, Vaginal bleeding, Acid reflux, Symphysis pubis dysfunction, Other | Yes or No  Pregnancy diabetes, Hyperthyroidism, Hypothyroidism, High blood pressure, Preeclampsia, Hyperemesis gravidarum, Depression, Vaginal bleeding, Acid reflux, Symphysis pubis dysfunction, Other |
| Alcohol during pregnancy | 1/month or less, 2-4/month, 2-3/week, 4/week or more, Never | No (Never) vs Yes (all groups except Never) |
| Drugs use during pregnancy | Prescription free pain medication, opioids/strong pain medication, allergy medication, asthma medication, antidepressant medication, anxiety medication, sleeping pills, thyroid hormones, blood pressure medication, stomach acid medication or other | (1) Asthma and allergy medication, (2) Anxiety, antidepressants and sleep medication, (3) Prescription free pain medication, (4) Opioids and strong pain medication, (5) Thyroid medication, (6) Blood pressure medication, (7) Stomach acid medication, (8) Other |
| Depression – Edinburgh postnatal depression scale (EPDS) | Questionnaire includes 10 standardized questions from the Edinburgh postnatal depression scale and scored according to guidelines | ≥13 and <13 |
| Perceived stress scale | Questionnaire includes 4 standardized questions from the Perceived stress scale and scored according to guidelines | Below 1^st^ quartile, 2^nd^-3^rd^ quartile, above 4th quartile |
| Bristol stool scale (BSS) | 7 standardized groups (scale 1-7) | Slow colonic transit (only BSS 1-4), Normal colonic transit (only BSS 3-4), Fast colonic transit (only BSS 3-7) and Various (BSS 1/2 and 5/6/7) |
| General health estimate | Very good, Pretty good, Neither good nor bad, Pretty bad, Very bad | Very good, Pretty good, Neither good nor bad, Pretty bad, Very bad |
| Vomiting in the last 24 hours | Yes or No | Yes or No |
| Nausea in the last 24 hours | None, Less than 1 hour, 2-3 hours, 4-6 hours, More than 6 hours | None, Less than 6 hours (Less than 1 hour, 2-3 hours, 4-6 hours), More than 6 hours (More than 6 hours) |

Supplementary Table 1: Overview of possible risk factors of pre-pregnancy complications

|  |  | **Total cohort (n=5330)** | | **Uncomplicated (n=4188)** | | **Complicated (n=1142)** | | **RPL (n=220)** | | **Subfertile (n=790)** | | **ART (n=540)** | | **Late miscarriage (n=96)** | |
| --- | --- | --- | --- | --- | --- | --- | --- | --- | --- | --- | --- | --- | --- | --- | --- |
|  |  | **n** | **%** | **n** | **%** | **n** | **%** | **n** | **%** | **n** | **%** | **n** | **%** | **n** | **%** |
| **Pregnancy week when answering** | Mean | 11.91 | | 11.90 | | 11.93 | | 12.21 | | 11.95 | | 12.01 | | 11.85 | |
| **Maternal age** | < 25 | 184 | 3.5 | 156 | 3.7 | 28 | 2.5 | 7 | 3.2 | 21 | 2.7 | 7 | 1.3 | 4 | 4.2 |
|  | 25 – 29 | 1444 | 27.1 | 1230 | 29.4 | 214 | 18.7 | 38 | 17.3 | 151 | 19.1 | 81 | 15.0 | 18 | 18.8 |
|  | 30 – 34 | 2450 | 46.0 | 1973 | 47.1 | 477 | 41.8 | 85 | 38.6 | 330 | 41.8 | 243 | 45.0 | 36 | 37.5 |
|  | ≥ 35 | 1252 | 23.5 | 829 | 19.8 | 423 | 37.0 | 90 | 40.9 | 288 | 36.5 | 209 | 38.7 | 38 | 39.6 |
| **Body mass index before pregnancy** | < 20 | 666 | 12.5 | 539 | 12.9 | 127 | 11.1 | 23 | 10.5 | 94 | 11.9 | 67 | 12.4 | 14 | 14.6 |
|  | 20 – 24.9 | 3063 | 57.5 | 2451 | 58.5 | 612 | 53.6 | 122 | 55.5 | 426 | 53.9 | 291 | 53.9 | 41 | 42.7 |
|  | 25 – 29.9 | 1091 | 20.5 | 827 | 19.7 | 264 | 23.1 | 51 | 23.2 | 169 | 21.4 | 136 | 25.2 | 21 | 21.9 |
|  | ≥ 30 | 493 | 9.2 | 360 | 8.6 | 133 | 11.6 | 23 | 10.5 | 99 | 12.5 | 44 | 8.1 | 18 | 18.8 |
|  | Missing | 17 | 0.3 | 11 | 0.3 | 6 | 0.5 | 1 | 0.5 | 2 | 0.3 | 2 | 0.4 | 2 | 2.1 |
| **Country of birth** | Sweden | 4819 | 90.4 | 3813 | 91.0 | 1006 | 88.1 | 192 | 87.3 | 689 | 87.2 | 483 | 89.4 | 83 | 86.5 |
|  | Other | 486 | 9.1 | 353 | 8.4 | 133 | 11.6 | 26 | 11.8 | 101 | 12.8 | 57 | 10.6 | 12 | 12.5 |
|  | Missing | 25 | 0.5 | 22 | 0.5 | 3 | 0.3 | 2 | 0.9 | 0 | 0.0 | 0 | 0.0 | 1 | 1.0 |
| **Highest level of education** | Primary school | 74 | 1.4 | 53 | 1.3 | 21 | 1.8 | 9 | 4.1 | 14 | 1.8 | 3 | 0.6 | 3 | 3.1 |
|  | Senior high school | 933 | 17.5 | 735 | 17.6 | 198 | 17.3 | 51 | 23.2 | 129 | 16.3 | 62 | 11.5 | 28 | 29.2 |
|  | University / college | 4145 | 77.8 | 3261 | 77.9 | 884 | 77.4 | 152 | 69.1 | 618 | 78.2 | 463 | 85.7 | 62 | 64.6 |
|  | Other | 177 | 3.3 | 138 | 3.3 | 39 | 3.4 | 8 | 3.6 | 29 | 3.7 | 12 | 2.2 | 3 | 3.1 |
|  | Missing | 1 | 0.0 | 1 | 0.0 | 0 | 0.0 | 0 | 0.0 | 0 | 0.0 | 0 | 0.0 | 0 | 0.0 |
| **Work situation** | Working | 4666 | 87.5 | 3650 | 87.2 | 1016 | 89.0 | 185 | 84.1 | 714 | 90.4 | 492 | 91.1 | 75 | 78.1 |
|  | Other | 663 | 12.4 | 537 | 12.8 | 126 | 11.0 | 35 | 15.9 | 76 | 9.6 | 48 | 8.9 | 21 | 21.9 |
|  | Missing | 1 | 0.0 | 1 | 0.0 | 0 | 0.0 | 0 | 0.0 | 0 | 0.0 | 0 | 0.0 | 0 | 0.0 |
| **First pregnancy** | Yes | 1493 | 28.0 | 1174 | 28.0 | 319 | 27.9 | 0 | 0.0 | 267 | 33.8 | 228 | 42.2 | 0 | 0.0 |
|  | No | 3837 | 72.0 | 3014 | 72.0 | 823 | 72.1 | 220 | 100.0 | 523 | 66.2 | 312 | 57.8 | 96 | 100.0 |
| **Contraceptives use the year before pregnancy** | Yes | 2276 | 42.7 | 2118 | 50.6 | 158 | 13.8 | 51 | 23.2 | 73 | 9.2 | 65 | 12.0 | 21 | 21.9 |
|  | No | 3054 | 57.3 | 2070 | 49.4 | 984 | 86.2 | 169 | 76.8 | 717 | 90.8 | 475 | 88.0 | 75 | 78.1 |
| **Contraceptives** | Hormonal | 1856 | 34.8 | 1733 | 41.4 | 123 | 10.8 | 37 | 16.8 | 64 | 8.1 | 53 | 9.8 | 13 | 13.5 |
|  | Not hormonal | 630 | 11.8 | 586 | 14.0 | 44 | 3.9 | 15 | 6.8 | 14 | 1.8 | 19 | 3.5 | 10 | 10.4 |
|  | Missing | 1 | 0.0 | 1 | 0.0 | 0 | 0.0 | 0 | 0.0 | 0 | 0.0 | 0 | 0.0 | 0 | 0.0 |
| **Regular menstruation** | Yes | 4399 | 82.5 | 3521 | 84.1 | 878 | 76.9 | 189 | 85.9 | 598 | 75.7 | 379 | 70.2 | 76 | 79.2 |
|  | No | 922 | 17.3 | 660 | 15.8 | 262 | 22.9 | 31 | 14.1 | 191 | 24.2 | 159 | 29.4 | 20 | 20.8 |
|  | Missing | 9 | 0.2 | 7 | 0.2 | 2 | 0.2 | 0 | 0.0 | 1 | 0.1 | 2 | 0.4 | 0 | 0.0 |
| **Endometriosis** | Yes | 202 | 3.8 | 98 | 2.3 | 104 | 9.1 | 16 | 7.3 | 78 | 9.9 | 64 | 11.9 | 4 | 4.2 |
|  | Maybe | 1100 | 20.6 | 841 | 20.1 | 259 | 22.7 | 54 | 24.5 | 193 | 24.4 | 120 | 22.2 | 25 | 26.0 |
|  | No | 4002 | 75.1 | 3229 | 77.1 | 773 | 67.7 | 150 | 68.2 | 517 | 65.4 | 353 | 65.4 | 66 | 68.8 |
|  | Missing | 26 | 0.5 | 20 | 0.5 | 6 | 0.5 | 0 | 0.0 | 2 | 0.3 | 3 | 0.6 | 1 | 1.0 |
| **Polycystic ovary syndrome** | Yes | 428 | 8.0 | 251 | 6.0 | 177 | 15.5 | 19 | 8.6 | 130 | 16.5 | 127 | 23.5 | 9 | 9.4 |
|  | Maybe | 260 | 4.9 | 201 | 4.8 | 59 | 5.2 | 12 | 5.5 | 45 | 5.7 | 24 | 4.4 | 4 | 4.2 |
|  | No | 4596 | 86.2 | 3694 | 88.2 | 902 | 79.0 | 187 | 85.0 | 613 | 77.6 | 389 | 72.0 | 82 | 85.4 |
|  | Missing | 46 | 0.9 | 42 | 1.0 | 4 | 0.4 | 2 | 0.9 | 2 | 0.3 | 0 | 0.0 | 1 | 1.0 |
| **Regular contact with animals** | Yes | 2683 | 50.3 | 2072 | 49.5 | 611 | 53.5 | 119 | 54.1 | 419 | 53.0 | 283 | 52.4 | 47 | 49.0 |
|  | No | 2645 | 49.6 | 2114 | 50.5 | 531 | 46.5 | 101 | 45.9 | 371 | 47.0 | 257 | 47.6 | 49 | 51.0 |
|  | Missing | 2 | 0.0 | 2 | 0.0 | 0 | 0.0 | 0 | 0.0 | 0 | 0.0 | 0 | 0.0 | 0 | 0.0 |
| **Ever attended cervical screening** | Yes | 4887 | 91.7 | 3833 | 91.5 | 1054 | 92.3 | 195 | 88.6 | 733 | 92.8 | 509 | 94.3 | 83 | 86.5 |
|  | No | 273 | 5.1 | 221 | 5.3 | 52 | 4.6 | 19 | 8.6 | 33 | 4.2 | 22 | 4.1 | 7 | 7.3 |
|  | Missing | 5 | 0.1 | 5 | 0.1 | 0 | 0.0 | 0 | 0.0 | 0 | 0.0 | 0 | 0.0 | 0 | 0.0 |
| **Ever had cell changes** | Yes | 1151 | 21.6 | 910 | 21.7 | 241 | 21.1 | 58 | 26.4 | 168 | 21.3 | 117 | 21.7 | 18 | 18.8 |
|  | No | 4050 | 76.0 | 3177 | 75.9 | 873 | 76.4 | 154 | 70.0 | 603 | 76.3 | 412 | 76.3 | 75 | 78.1 |
|  | Missing | 7 | 0.1 | 7 | 0.2 | 0 | 0.0 | 0 | 0.0 | 0 | 0.0 | 0 | 0.0 | 0 | 0.0 |
| **Smoking habits** | Current smoker | 67 | 1.3 | 52 | 1.2 | 15 | 1.3 | 10 | 4.5 | 5 | 0.6 | 3 | 0.6 | 4 | 4.2 |
|  | Other | 2044 | 38.3 | 1593 | 38.0 | 451 | 39.5 | 98 | 44.5 | 305 | 38.6 | 199 | 36.9 | 44 | 45.8 |
|  | Never | 3205 | 60.1 | 2533 | 60.5 | 672 | 58.8 | 111 | 50.5 | 478 | 60.5 | 335 | 62.0 | 48 | 50.0 |
|  | Missing | 14 | 0.3 | 10 | 0.2 | 4 | 0.4 | 1 | 0.5 | 2 | 0.3 | 3 | 0.6 | 0 | 0.0 |
| **Mouth tobacco habits** | Current user | 66 | 1.2 | 49 | 1.2 | 17 | 1.5 | 1 | 0.5 | 14 | 1.8 | 6 | 1.1 | 2 | 2.1 |
|  | Other | 1133 | 21.3 | 886 | 21.2 | 247 | 21.6 | 46 | 20.9 | 173 | 21.9 | 123 | 22.8 | 19 | 19.8 |
|  | Never | 4107 | 77.1 | 3234 | 77.2 | 873 | 76.4 | 170 | 77.3 | 601 | 76.1 | 409 | 75.7 | 74 | 77.1 |
|  | Missing | 24 | 0.5 | 19 | 0.5 | 5 | 0.4 | 3 | 1.4 | 2 | 0.3 | 2 | 0.4 | 1 | 1.0 |
| **Diagnosed eating disorder** | Yes | 525 | 9.8 | 404 | 9.6 | 121 | 10.6 | 28 | 12.7 | 82 | 10.4 | 55 | 10.2 | 11 | 11.5 |
|  | No | 4733 | 88.8 | 3730 | 89.1 | 1003 | 87.8 | 184 | 83.6 | 699 | 88.5 | 479 | 88.7 | 83 | 86.5 |
|  | Missing | 6 | 0.1 | 5 | 0.1 | 1 | 0.1 | 0 | 0.0 | 1 | 0.1 | 1 | 0.2 | 0 | 0.0 |
| **Gynecological infections in the last 3 months** | Sexually transmitted diseases (STDs) | 285 | 5.3 | 226 | 5.4 | 59 | 5.2 | 6 | 2.7 | 43 | 5.4 | 33 | 6.1 | 3 | 3.1 |
|  | Missing | 44 | 0.8 | 32 | 0.8 | 12 | 1.1 | 1 | 0.5 | 9 | 1.1 | 5 | 0.9 | 2 | 2.1 |
|  | Bacterial vaginosis | 203 | 3.8 | 158 | 3.8 | 45 | 3.9 | 12 | 5.5 | 32 | 4.1 | 23 | 4.3 | 4 | 4.2 |
|  | Missing | 26 | 0.5 | 23 | 0.5 | 3 | 0.3 | 1 | 0.5 | 1 | 0.1 | 2 | 0.4 | 0 | 0.0 |
|  | Fungal infection | 914 | 17.1 | 722 | 17.2 | 192 | 16.8 | 44 | 20.0 | 133 | 16.8 | 95 | 17.6 | 20 | 20.8 |
|  | Missing | 20 | 0.4 | 14 | 0.3 | 6 | 0.5 | 1 | 0.5 | 4 | 0.5 | 2 | 0.4 | 0 | 0.0 |
|  | *Mycoplasma genitalium* | 12 | 0.2 | 10 | 0.2 | 2 | 0.2 | 0 | 0.0 | 2 | 0.3 | 1 | 0.2 | 0 | 0.0 |
|  | Missing | 33 | 0.6 | 26 | 0.6 | 7 | 0.6 | 3 | 1.4 | 3 | 0.4 | 4 | 0.7 | 1 | 1.0 |
|  | Condyloma | 99 | 1.9 | 78 | 1.9 | 21 | 1.8 | 3 | 1.4 | 14 | 1.8 | 9 | 1.7 | 3 | 3.1 |
|  | Missing | 29 | 0.5 | 23 | 0.5 | 6 | 0.5 | 1 | 0.5 | 4 | 0.5 | 3 | 0.6 | 1 | 1.0 |
|  | Other | 63 | 1.2 | 46 | 1.1 | 17 | 1.5 | 3 | 1.4 | 10 | 1.3 | 9 | 1.7 | 4 | 4.2 |
|  | Missing | 53 | 1.0 | 46 | 1.1 | 7 | 0.6 | 2 | 0.9 | 5 | 0.6 | 2 | 0.4 | 0 | 0.0 |
|  | Any infection | 1240 | 23.3 | 972 | 23.2 | 268 | 23.5 | 51 | 23.2 | 185 | 23.4 | 133 | 24.6 | 27 | 28.1 |
|  | Missing | 52 | 1.0 | 38 | 0.9 | 14 | 1.2 | 1 | 0.5 | 12 | 1.5 | 3 | 0.6 | 1 | 1.0 |
| **Drug use before pregnancy** | Asthma and allergy medication | 834 | 15.6 | 630 | 15.0 | 204 | 17.9 | 46 | 20.9 | 143 | 18.1 | 78 | 14.4 | 16 | 16.7 |
|  | Anxiety, antidepressants, and sleep medication | 687 | 12.9 | 525 | 12.5 | 162 | 14.2 | 37 | 16.8 | 117 | 14.8 | 73 | 13.5 | 15 | 15.6 |
|  | Prescription free pain medication | 1397 | 26.2 | 1074 | 25.6 | 323 | 28.3 | 62 | 28.2 | 234 | 29.6 | 147 | 27.2 | 20 | 20.8 |
|  | Opioids and strong pain medication | 72 | 1.4 | 47 | 1.1 | 25 | 2.2 | 8 | 3.6 | 18 | 2.3 | 13 | 2.4 | 3 | 3.1 |
|  | Thyroid medication | 373 | 7.0 | 239 | 5.7 | 134 | 11.7 | 29 | 13.2 | 84 | 10.6 | 68 | 12.6 | 10 | 10.4 |
|  | Blood pressure medication | 45 | 0.8 | 33 | 0.8 | 12 | 1.1 | 4 | 1.8 | 10 | 1.3 | 6 | 1.1 | 1 | 1.0 |
|  | Stomach acid medication | 301 | 5.6 | 227 | 5.4 | 74 | 6.5 | 17 | 7.7 | 56 | 7.1 | 36 | 6.7 | 7 | 7.3 |
|  | Other | 475 | 8.9 | 363 | 8.7 | 112 | 9.8 | 22 | 10.0 | 82 | 10.4 | 57 | 10.6 | 11 | 11.5 |
|  | Missing | 52 | 1.0 | 41 | 1.0 | 11 | 1.0 | 1 | 0.5 | 8 | 1.0 | 6 | 1.1 | 1 | 1.0 |
| **Vegetarian** | Yes | 927 | 17.4 | 731 | 17.5 | 196 | 17.2 | 19 | 8.6 | 133 | 16.8 | 114 | 21.1 | 12 | 12.5 |
|  | No | 4395 | 82.5 | 3451 | 82.4 | 944 | 82.7 | 200 | 90.9 | 655 | 82.9 | 425 | 78.7 | 84 | 87.5 |
|  | Missing | 8 | 0.2 | 6 | 0.1 | 2 | 0.2 | 1 | 0.5 | 2 | 0.3 | 1 | 0.2 | 0 | 0.0 |
| **Eats fish** | Yes | 4815 | 90.3 | 3780 | 90.3 | 1035 | 90.6 | 206 | 93.6 | 723 | 91.5 | 482 | 89.3 | 88 | 91.7 |
|  | No | 513 | 9.6 | 406 | 9.7 | 107 | 9.4 | 14 | 6.4 | 67 | 8.5 | 58 | 10.7 | 8 | 8.3 |
|  | Missing | 2 | 0.0 | 2 | 0.0 | 0 | 0.0 | 0 | 0.0 | 0 | 0.0 | 0 | 0.0 | 0 | 0.0 |
| **Sweet drinks consumed** | Often | 1873 | 35.1 | 1467 | 35.0 | 406 | 35.6 | 83 | 37.7 | 293 | 37.1 | 183 | 33.9 | 32 | 33.3 |
|  | Seldom | 3456 | 64.8 | 2720 | 64.9 | 736 | 64.4 | 137 | 62.3 | 497 | 62.9 | 357 | 66.1 | 64 | 66.7 |
|  | Missing | 1 | 0.0 | 1 | 0.0 | 0 | 0.0 | 0 | 0.0 | 0 | 0.0 | 0 | 0.0 | 0 | 0.0 |
| **Sugarfree drinks consumed** | Often | 1133 | 21.3 | 893 | 21.3 | 240 | 21.0 | 43 | 19.5 | 165 | 20.9 | 119 | 22.0 | 18 | 18.8 |
|  | Seldom | 4183 | 78.5 | 3284 | 78.4 | 899 | 78.7 | 177 | 80.5 | 623 | 78.9 | 418 | 77.4 | 78 | 81.3 |
|  | Missing | 14 | 0.3 | 11 | 0.3 | 3 | 0.3 | 0 | 0.0 | 2 | 0.3 | 3 | 0.6 | 0 | 0.0 |
| **Wholegrain bread consumed** | Often | 2142 | 40.2 | 1699 | 40.6 | 443 | 38.8 | 84 | 38.2 | 299 | 37.8 | 219 | 40.6 | 41 | 42.7 |
|  | Seldom | 3183 | 59.7 | 2486 | 59.4 | 697 | 61.0 | 136 | 61.8 | 490 | 62.0 | 320 | 59.3 | 54 | 56.3 |
|  | Missing | 5 | 0.1 | 3 | 0.1 | 2 | 0.2 | 0 | 0.0 | 1 | 0.1 | 1 | 0.2 | 1 | 1.0 |
| **Fruit consumed** | Often | 3906 | 73.3 | 3075 | 73.4 | 831 | 72.8 | 156 | 70.9 | 570 | 72.2 | 399 | 73.9 | 66 | 68.8 |
|  | Seldom | 1422 | 26.7 | 1111 | 26.5 | 311 | 27.2 | 64 | 29.1 | 220 | 27.8 | 141 | 26.1 | 30 | 31.3 |
|  | Missing | 2 | 0.0 | 2 | 0.0 | 0 | 0.0 | 0 | 0.0 | 0 | 0.0 | 0 | 0.0 | 0 | 0.0 |
| **Vegetables consumed** | Often | 3947 | 74.1 | 3088 | 73.7 | 859 | 75.2 | 158 | 71.8 | 594 | 75.2 | 417 | 77.2 | 76 | 79.2 |
|  | Seldom | 1383 | 25.9 | 1100 | 26.3 | 283 | 24.8 | 62 | 28.2 | 196 | 24.8 | 123 | 22.8 | 20 | 20.8 |
|  | Missing | 0 | 0.0 | 0 | 0 | 0 | 0 | 0 | 0 | 0 | 0 | 0 | 0 | 0 | 0 |

RPL, Recurrent pregnancy loss; ART, Artificial reproductive technologies

Supplementary Table 2: Multivariable logistic regression to assess risk factors of pre-pregnancy complications, with significant results in bold font; expressed as odds ratios (OR) and 95% confidence intervals (CI).

|  |  | **Complicated (n=1142)** | **RPL (n=220)** | **Subfertile (n=790)** | **ART (n=540)** |
| --- | --- | --- | --- | --- | --- |
| **Maternal age** | < 25 | reference | reference | reference | reference |
|  | 25 – 29 | 0.97 CI 0.64-1.51 | 0.69 CI 0.32-1.71 | 0.91 CI 0.57-1.52 | 1.48 CI 0.72-3.57 |
|  | 30 – 34 | 1.35 CI 0.90-2.08 | 0.96 CI 0.47-2.32 | 1.24 CI 0.79-2.04 | **2.77 CI 1.38-5.59** |
|  | ≥ 35 | **2.84 CI 1.90-4.40** | **2.42 CI 1.18-5.84** | **2.58 CI 1.64-4.26** | **5.67 CI 2.82-13.51** |
| **Body mass index before pregnancy** | < 20 | reference | reference | reference | reference |
|  | 20 – 24.9 | 1.06 CI 0.86-1.31 | 1.06 CI 0.86-1.32 | 1.06 CI 0.86-1.32 | 1.06 CI 0.86-1.32 |
|  | 25 – 29.9 | **1.35 CI 1.07-1.72** | **1.35 CI 1.07-1.72** | **1.35 CI 1.07-1.72** | **1.35 CI 1.07-1.72** |
|  | ≥ 30 | **1.58 CI 1.19-2.07** | **1.57 CI 1.19-2.07** | **1.57 CI 1.19-2.07** | **1.57 CI 1.19-2.07** |
| **Country of birth** | Outside Sweden | **1.42 CI 1.15-1-76** | 1.46 CI 0.94-2.19 | **1.58 CI 1.24-2.00** | 1.27 CI 0.94-1.69 |
| **Highest level of education** | Primary school | 1.46 CI 0.86-2.40 | **3.64 CI 1.65-7.17** | 1.39 CI 0.74-2.46 | 0.40 CI 0.10-1.08 |
|  | Senior high school | 0.99 CI 0.83-1.18 | **1.49 CI 1.06-2.05** | 0.93 CI 0.75-1.13 | **0.59 CI 0.45-0.78** |
|  | University / college | reference | reference | reference | reference |
|  | Other | 1.04 CI 0.72-1.48 | 1.24 CI 0.55-2.42 | 1.11 CI 0.72-1.64 | 0.62 CI 0.32-1.08 |
| **Work situation** | Not working full/part time | 0.84 CI 0.68-1.03 | 1.29 CI 0.87-1.82 | **0.72 CI 0.56-0.93** | **0.66 CI 0.48-0.89** |
| **First pregnancy** | Yes | 1.00 CI 0.86-1.15 | NA | **1.31 CI 1.11-1.54** | **1.88 CI 1.56-2.26** |
| **Contraceptives use the year before pregnancy** | No | **6.37 CI 5.35-7.64** | **3.39 CI 2.48-4.71** | **10.05 CI 7.90-12.98** | **7.50 CI 5.79-9.87** |
| **Contraceptives** | Hormonal | 0.78 CI 0.53-1.17 | 0.59 CI 0.32-1.13 | 1.58 CI 0.82-3.42 | 0.98 CI 0.54-1.94 |
|  | Not hormonal | 1.01 CI 0.70-1.43 | 1.09 CI 0.57-1.96 | 0.62 CI 0.33-1.09 | 1.08 CI 0.61-1.83 |
| **Regular menstruation** | Not regular menstruation | **1.59 CI 1.35-1.87** | 0.87 CI 0.58-1.27 | **1.70 CI 1.42-2.04** | **2.29 CI 1.87-2.80** |
| **Endometriosis** | Yes | **4.43 CI 3.33-5.91** | **3.51 CI 1.95-5.95** | **4.97 CI 3.63-6.78** | **6.06 CI 4.32-8.44** |
|  | Maybe | **1.29 CI 1.09-1.51** | **1.38 CI 1.00-1.89** | **1.43 CI 1.19-1.72** | **1.31 CI 1.04-1.62** |
| **Polycystic ovary syndrome** | Yes | **2.89 CI 2.35-3.54** | 1.50 CI 0.89-2.38 | **3.12 CI 2.48-3.92** | **5.13 CI 4.03-6.52** |
|  | Maybe | 1.20 CI 0.88-1.61 | 1.18 CI 0.61-2.06 | 1.35 CI 0.96-1.87 | 1.14 CI 0.72-1.72 |
| **Contact with animals** | Yes | **1.17 CI 1.03-1.34** | 1.20 CI 0.92-1.58 | 1.15 CI 0.99-1.34 | 1.12 CI 0.93-1.34 |
| **Ever attended cervical screening** | No | 0.86 CI 0.62-1.16 | **1.69 CI 1.00-2.69** | 0.78 CI 0.53-1.12 | 0.75 CI 0.47-1.15 |
| **Ever had cell changes** | Yes | 0.96 CI 0.82-1.13 | 1.31 CI 0.96-1.78 | 0.97 CI 0.81-1.17 | 1.00 CI 0.80-1.24 |
| **Smoking** | Current smoker | 1.09 CI 0.59-1.89 | **4.39 CI 2.06-8.51** | 0.51 CI 0.18-1.16 | 0.44 CI 0.11-1.21 |
|  | Other | 1.06 CI 0.93-1.22 | **1.40 CI 1.06-1.85** | 1.01 CI 0.87-1.19 | 0.95 CI 0.79-1.14 |
| **Mouth tobacco** | Current user | 1.29 CI 0.72-2.20 | 0.39 CI 0.02-1.79 | 1.54 CI 0.81-2.73 | 0.96 CI 0.37-2.09 |
|  | Other | 1.03 CI 0.88-1.21 | 0.99 CI 0.70-1.37 | 1.05 CI 0.87-1.26 | 1.10 CI 0.89-1.36 |
| **Diagnosed eating disorder** | Yes | 1.11 CI 0.90-1.38 | 1.40 CI 0.91-2.08 | 1.08 CI 0.84-1.38 | 1.06 CI 0.78-1.42 |
| **Gynecological infections** | Sexually transmitted diseases (STDs) | 0.96 CI 0.71-1.28 | 0.49 CI 0.19-1.02 | 1.01 CI 0.72-1.40 | 1.15 CI 0.78-1.65 |
|  | Bacterial vaginosis | 1.04 CI 0.74-1.45 | 1.47 CI 0.76-2.58 | 1.07 CI 0.72-1.56 | 1.15 CI 0.71-1.76 |
|  | Fungal infection | 0.97 CI 0.81-1.16 | 1.20 CI 0.85-1.67 | 0.97 CI 0.80-1.19 | 1.03 CI 0.81-1.29 |
|  | *Mycoplasma genitalium* | 0.73 CI 0.11-2.79 | NA | 1.06 CI 0.16-4.02 | 0.77 CI 0.04-4.03 |
|  | Condyloma | 0.99 CI 0.59-1.57 | 0.73 CI 0.18-1.97 | 0.95 CI 0.51-1.63 | 0.87 CI 0.41-1.68 |
|  | Other | 1.35 CI 0.75-2.32 | 1.24 CI 0.30-3.43 | 1.15 CI 0.54-2.19 | 1.50 CI 0.68-2.94 |
|  | Any infection | 1.02 CI 0.87-1.19 | 0.99 CI 0.71-1.83 | 1.02 CI 0.85-1.22 | 1.08 CI 0.87-1.33 |
| **Drug use before pregnancy** | Asthma and allergy medication | **1.23 CI 1.03-1.46** | **1.48 CI 1.05-2.06** | **1.25 CI 1.02-1.52** | 0.96 CI 0.74-1.23 |
|  | Anxiety. antidepressants and sleep medication | 1.15 CI 0.95-1.39 | 1.40 CI 0.96-2.00 | 1.21 CI 0.97-1.50 | 1.10 CI 0.84-1.42 |
|  | Prescription free pain medication | 1.14 CI 0.99-1.32 | 1.13 CI 0.83-1.52 | **1.22 CI 1.03-1.44** | 1.09 CI 0.89-1.33 |
|  | Opioids and strong pain medication | **1.97 CI 1.19 - 3.19** | **3.31 CI 1.43-6.71** | **2.06 CI 1.16-3.49** | **2.26 CI 1.16-4.09** |
|  | Thyroid medication | **2.20 CI 1.76-2.74** | **2.50 CI 1.62-3.71** | **1.97 CI 1.51-2.56** | **2.40 CI 1.79-3.18** |
|  | Blood pressure medication | 1.34 CI 0.66-2.53 | 2.32 CI 0.69-5.90 | 1.61 CI 0.75-3.17 | 1.45 CI 0.54-3.24 |
|  | Stomach acid medication | 1.21 CI 0.92-1.58 | 1.45 CI 0.84-2.36 | 1.33 CI 0.98-1.79 | 1.26 CI 0.86-1.78 |
|  | Other | 1.15 CI 0.91-1.43 | 1.16 CI 0.72-1.79 | 1.22 CI 0.94-1.56 | 1.25 CI 0.92-1.67 |
| **Vegetarian** | Yes | 0.98 CI 0.82-1.16 | **0.45 CI 0.27-0.70** | 0.96 CI 0.78-1.17 | **1.27 CI 1.01-1.58** |
| **Eats fish** | Yes | 1.04 CI 0.83-1.30 | 1.58 CI 0.95-2.87 | 1.16 CI 0.89-1.53 | 0.89 CI 0.67-1.20 |
| **Drinks sugary drinks** | Often | 1.02 CI 0.89-1.17 | 1.12 CI 0.85-1.48 | 1.09 CI 0.93-1.28 | 0.95 CI 0.79-1.15 |
| **Drinks sugarfree drinks** | Often | 0.98 CI 0.83-1.15 | 0.89 CI 0.63-1.24 | 0.97 CI 0.81-1.17 | 1.05 CI 0.84-1.30 |
| **Eats wholegrain bread** | Often | 0.93 CI 0.81-1.06 | 0.90 CI 0.68-1.19 | 0.89 CI 0.76-1.04 | 1.00 CI 0.83-1.20 |
| **Eats fruit** | Often | 0.97 CI 0.83-1.12 | 0.88 CI 0.66-1.19 | 0.94 CI 0.79-1.11 | 1.02 CI 0.84-1.26 |
| **Eats vegetables** | Often | 1.08 CI 0.93-1.26 | 0.91 CI 0.68-1.24 | 1.08 CI 0.91-1.29 | 1.21 CI 0.98-1.50 |

RPL, Recurrent pregnancy loss; ART, Artificial reproductive technologies

Supplementary Table 3: Multivariable logistic regression to assess if comorbidities are risk factors of pre-pregnancy complications with significant results in bold font. Not having the specific comorbidity was used as reference.

|  | **Complicated (n=1142)** | **RPL (n=220)** | **Subfertile (n=790)** | **ART (n=540)** |
| --- | --- | --- | --- | --- |
| **Gynaecological** | **OR 1.96, CI 1.71-2.24** | **OR 1.67, CI 1.27-2.20** | **OR 2.26, CI 1.94-2.64** | **OR 2.68, CI 2.23-3.21** |
| **Chronic inflammatory diseases** | OR 0.98, CI 0.62-1.51 | OR 1.02, CI 0.36-2.29 | OR 1.02, CI 0.60-1.66 | OR 0.92, CI 0.46-1.65 |
| **Gastrointestinal** | OR 1.10, CI 0.83-1.44 | OR 1.52, CI 0.89-2.43 | OR 1.20, CI 0.87-1.62 | OR 0.83, CI 0.53-1.24 |
| **Mental health** | OR 1.16, CI 0.97-1.37 | OR 1.27, CI 0.89-1.87 | OR 1.12, CI 0.92-1.37 | OR 1.13, CI 0.89-1.42 |
| **Chronic respiratory diseases and allergies** | OR 1.03, CI 0.89-1.19 | **OR 1.36, CI 1.02-1.80** | OR 1.03, CI 0.87-1.21 | OR 0.87, CI 0.71-1.06 |
| **Endocrine** | **OR 1.70, CI 1.40-2.06** | **OR 1.80, CI 1.22-2.59** | **OR 1.60, CI 1.27-1.99** | **OR 1.86, CI 1.44-2.38** |
| **Any comorbidity** | **OR 1.69, CI 1.46-1.97** | **OR 1.85, CI 1.35-2.56** | **OR 1.76, CI 1.48-2.10** | **OR 2.06, CI 1.67-2.56** |
| **No comorbidity** | **OR 0.59, CI 0.51-0.68** | **OR 0.54, CI 0.39-0.74** | **OR 0.57, CI 0.48-0.67** | **OR 0.49, CI 0.39-0.60** |

*RPL, Recurrent pregnancy loss; Artificial reproductive technologies.

Supplementary Table 4: Well-being in early pregnancy by pre-pregnancy complications, and it’s subgroups.

|  |  | **Total cohort (n=5330)** | | **Uncomplicated (n=4188)** | | **Complicated (n=1142)** | | **RPL (n=220)** | | **Subfertile (n=790)** | | **ART (n=540)** | | **Late miscarriage (n=96)** | |
| --- | --- | --- | --- | --- | --- | --- | --- | --- | --- | --- | --- | --- | --- | --- | --- |
|  |  | **n** | **%** | **n** | **%** | **n** | **%** | **n** | **%** | **n** | **%** | **n** | **%** | **n** | **%** |
| **Perceived stress (PSS)** | Mean | 5.43 | | 5.43 | | 5.42 | | 5.77 | | 5.45 | | 5.3 | | 5.86 | |
|  | < 4 | 1296 | 24.3 | 1013 | 24.2 | 280 | 24.5 | 52 | 23.6 | 191 | 24.2 | 141 | 26.1 | 21 | 21.9 |
|  | 4-7 | 2207 | 41.4 | 1742 | 41.6 | 467 | 40.9 | 82 | 37.3 | 322 | 40.8 | 225 | 41.7 | 31 | 32.3 |
|  | > 7 | 1830 | 34.3 | 1433 | 34.2 | 395 | 34.6 | 86 | 39.1 | 277 | 35.1 | 174 | 32.2 | 44 | 45.8 |
|  | Missing | 0 | 0.0 | 0 | 0.0 | 0 | 0.0 | 0 | 0.0 | 0 | 0.0 | 0 | 0.0 | 0 | 0.0 |
| **Depression (EPDS)** | Mean | 7.06 | | 7.02 | | 7.22 | | 8.17 | | 7.26 | | 6.7 | | 8.71 | |
|  | < 13 | 4613 | 86.5 | 3641 | 86.9 | 971 | 85.0 | 175 | 78.6 | 677 | 84.9 | 473 | 86.7 | 73 | 60.4 |
|  | ≥ 13 | 718 | 13.5 | 547 | 13.1 | 171 | 15.0 | 45 | 22.3 | 113 | 15.6 | 67 | 13.9 | 23 | 21.9 |
|  | Missing | 0 | 0.0 | 0 | 0.0 | 0 | 0.0 | 0 | 0.0 | 0 | 0.0 | 0 | 0.0 | 0 | 0.0 |
| **Bristol scale (BSS)** | Slow colonic transit (only BSS 1-4) | 1530 | 28.7 | 1184 | 28.3 | 345 | 30.2 | 77 | 35.0 | 241 | 30.5 | 161 | 29.8 | 28 | 29.2 |
|  | Normal colonic transit (only BSS 3-4) | 1083 | 20.3 | 857 | 20.5 | 226 | 19.8 | 45 | 20.5 | 142 | 18.0 | 114 | 21.1 | 22 | 22.9 |
|  | Fast colonic transit (only BSS 3-7) | 1360 | 25.5 | 1088 | 26.0 | 272 | 23.8 | 47 | 21.4 | 193 | 24.4 | 130 | 24.1 | 27 | 28.1 |
|  | Various (BSS 1/2 and 5/6/7) | 1345 | 25.2 | 1047 | 25.0 | 297 | 26.0 | 51 | 23.2 | 213 | 27.0 | 134 | 24.8 | 18 | 18.8 |
|  | Missing | 14 | 0.3 | 12 | 0.3 | 2 | 0.2 | 0 | 0.0 | 1 | 0.1 | 1 | 0.2 | 1 | 1.0 |
| **Pregnancy problems** | No | 4569 | 85.7 | 3622 | 86.5 | 947 | 82.9 | 165 | 75.0 | 650 | 82.3 | 447 | 82.8 | 81 | 84.4 |
|  | Yes | 754 | 14.1 | 559 | 13.3 | 195 | 17.1 | 55 | 25.0 | 140 | 17.7 | 93 | 17.2 | 15 | 15.6 |
|  | Pregnancy diabetes | 11 | 0.2 | 7 | 0.2 | 4 | 0.4 | 2 | 0.9 | 2 | 0.3 | 1 | 0.2 | 2 | 2.1 |
|  | Hyperthyroidism | 28 | 0.5 | 19 | 0.5 | 9 | 0.8 | 5 | 2.3 | 2 | 0.3 | 3 | 0.6 | 0 | 0.0 |
|  | Hypothyroidism | 107 | 2.0 | 73 | 1.7 | 34 | 3.0 | 8 | 3.6 | 24 | 3.0 | 21 | 3.9 | 1 | 1.0 |
|  | High blood pressure | 21 | 0.4 | 17 | 0.4 | 4 | 0.4 | 3 | 1.4 | 4 | 0.5 | 1 | 0.2 | 0 | 0.0 |
|  | Preeclampsia | 5 | 0.1 | 5 | 0.1 | 0 | 0.0 | 0 | 0.0 | 0 | 0.0 | 0 | 0.0 | 0 | 0.0 |
|  | Hyperemesis gravidarum | 150 | 2.8 | 104 | 2.5 | 46 | 4.0 | 11 | 5.0 | 34 | 4.3 | 18 | 3.3 | 3 | 3.1 |
|  | Depression | 84 | 1.6 | 55 | 1.3 | 29 | 2.5 | 9 | 4.1 | 17 | 2.2 | 11 | 2.0 | 6 | 6.3 |
|  | Vaginal bleeding | 138 | 2.6 | 90 | 2.1 | 48 | 4.2 | 18 | 8.2 | 32 | 4.1 | 24 | 4.4 | 3 | 3.1 |
|  | Acid reflux | 236 | 4.4 | 170 | 4.1 | 66 | 5.8 | 18 | 8.2 | 52 | 6.6 | 31 | 5.7 | 6 | 6.3 |
|  | Symphysis pubis dysfunction | 183 | 3.4 | 137 | 3.3 | 46 | 4.0 | 20 | 9.1 | 36 | 4.6 | 19 | 3.5 | 3 | 3.1 |
|  | Other | 311 | 5.8 | 237 | 5.7 | 74 | 6.5 | 16 | 7.3 | 58 | 7.3 | 38 | 7.0 | 2 | 2.1 |
|  | Missing | 7 | 0.1 | 7 | 0.2 | 0 | 0.0 | 0 | 0.0 | 0 | 0.0 | 0 | 0.0 | 0 | 0.0 |
| **Vomiting in the last 24 hours** | Yes | 547 | 10.3 | 427 | 10.2 | 120 | 10.5 | 29 | 13.2 | 81 | 10.3 | 43 | 8.0 | 13 | 13.5 |
|  | No | 4775 | 89.6 | 3756 | 89.7 | 1019 | 89.2 | 190 | 86.4 | 706 | 89.4 | 496 | 91.9 | 83 | 86.5 |
|  | Missing | 8 | 0.2 | 5 | 0.1 | 3 | 0.3 | 1 | 0.5 | 3 | 0.4 | 1 | 0.2 | 0 | 0.0 |
| **Nausea in the last 24 hours** | None | 1647 | 30.9 | 1272 | 30.4 | 375 | 32.8 | 59 | 26.8 | 272 | 34.4 | 180 | 33.3 | 26 | 27.1 |
|  | Less than 6 hours | 2856 | 53.6 | 2267 | 54.1 | 589 | 51.6 | 122 | 55.5 | 395 | 50.0 | 283 | 52.4 | 50 | 52.1 |
|  | More than 6 hours | 818 | 15.3 | 645 | 15.4 | 173 | 15.1 | 36 | 16.4 | 119 | 15.1 | 74 | 13.7 | 19 | 19.8 |
|  | Missing | 9 | 0.2 | 4 | 0.1 | 5 | 0.4 | 3 | 1.4 | 4 | 0.5 | 3 | 0.6 | 1 | 1.0 |
| **General health (self-estimate)** | Very good | 2126 | 39.9 | 1700 | 40.6 | 426 | 37.3 | 60 | 27.3 | 294 | 37.2 | 220 | 40.7 | 29 | 30.2 |
|  | Pretty good | 2752 | 51.6 | 2152 | 51.4 | 600 | 52.5 | 122 | 55.5 | 424 | 53.7 | 284 | 52.6 | 53 | 55.2 |
|  | Neither good nor bad | 367 | 6.9 | 268 | 6.4 | 99 | 8.7 | 33 | 15.0 | 60 | 7.6 | 32 | 5.9 | 11 | 11.5 |
|  | Pretty bad | 75 | 1.4 | 60 | 1.4 | 15 | 1.3 | 5 | 2.3 | 10 | 1.3 | 2 | 0.4 | 3 | 3.1 |
|  | Very bad | 7 | 0.1 | 5 | 0.1 | 2 | 0.2 | 0 | 0.0 | 2 | 0.3 | 2 | 0.4 | 0 | 0.0 |
|  | Missing | 3 | 0.1 | 3 | 0.1 | 0 | 0.0 | 0 | 0.0 | 0 | 0.0 | 0 | 0.0 | 0 | 0.0 |
| **Alcohol while pregnant** | Yes | 347 | 6.5 | 295 | 7.0 | 52 | 4.6 | 7 | 3.2 | 41 | 5.2 | 13 | 2.4 | 5 | 5.2 |
|  | No | 4980 | 93.4 | 3890 | 92.9 | 1090 | 95.4 | 213 | 96.8 | 749 | 94.8 | 527 | 97.6 | 91 | 94.8 |
|  | Missing | 3 | 0.1 | 3 | 0.1 | 0 | 0.0 | 0 | 0.0 | 0 | 0.0 | 0 | 0.0 | 0 | 0.0 |
| **Medication during pregnancy** | Yes | 2043 | 38.3 | 1527 | 36.5 | 516 | 45.2 | 122 | 55.5 | 350 | 44.3 | 237 | 43.9 | 50 | 52.1 |
|  | No | 3224 | 60.5 | 2608 | 62.3 | 616 | 53.9 | 97 | 44.1 | 434 | 54.9 | 297 | 55.0 | 46 | 47.9 |
|  | Missing | 63 | 1.2 | 53 | 1.3 | 10 | 0.9 | 1 | 0.5 | 6 | 0.8 | 6 | 1.1 | 0 | 0.0 |
| **Drug use during pregnancy** | Asthma and allergy medication | 437 | 8.2 | 323 | 7.7 | 114 | 10.0 | 29 | 13.2 | 77 | 9.7 | 44 | 8.1 | 10 | 10.4 |
|  | Anxiety, antidepressants, and sleep medication | 434 | 8.1 | 329 | 7.9 | 105 | 9.2 | 21 | 9.5 | 73 | 9.2 | 49 | 9.1 | 9 | 9.4 |
|  | Prescription free pain medication | 520 | 9.8 | 410 | 9.8 | 110 | 9.6 | 29 | 13.2 | 76 | 9.6 | 38 | 7.0 | 10 | 10.4 |
|  | Opioids and strong pain medication | 19 | 0.4 | 9 | 0.2 | 10 | 0.9 | 3 | 1.4 | 7 | 0.9 | 5 | 0.9 | 3 | 3.1 |
|  | Thyroid medication | 444 | 8.3 | 298 | 7.1 | 146 | 12.8 | 32 | 14.5 | 95 | 12.0 | 78 | 14.4 | 13 | 13.5 |
|  | Blood pressure medication | 35 | 0.7 | 24 | 0.6 | 11 | 1.0 | 6 | 2.7 | 9 | 1.1 | 5 | 0.9 | 1 | 1.0 |
|  | Stomach acid medication | 252 | 4.7 | 189 | 4.5 | 63 | 5.5 | 16 | 7.3 | 47 | 5.9 | 23 | 4.3 | 7 | 7.3 |
|  | Other | 583 | 10.9 | 420 | 10.0 | 163 | 14.3 | 44 | 20.0 | 107 | 13.5 | 78 | 14.4 | 26 | 27.1 |
|  | Missing | 63 | 1.2 | 53 | 1.3 | 10 | 0.9 | 1 | 0.5 | 6 | 0.8 | 6 | 1.1 | 0 | 0.0 |

RPL, Recurrent pregnancy loss; ART, Artificial reproductive technologies; EDPS, Edinburgh Postnatal Depression Scale.

Supplementary Table 4: Multivariable logistic regression showing odds ratio (OR) and 95% confidence intervals for pregnancy symptoms for pre-pregnancy complications and its subgroups, with an uncomplicated pre-pregnancy onset as a reference. Significant results in bold font.

|  |  | **Complicated (n=1142)** | **RPL (n=220)** | **Subfertile (n=790)** | **ART (n=540)** |
| --- | --- | --- | --- | --- | --- |
| **Perceived stress (PSS)** | > 7 | 0.93, CI 0.74-1.16 | 1.26, CI 0.92-1.70 | 1.06, CI 0.88-1.26 | 0.92, CI 0.74-1.16 |
| **Depression (EPDS)** | ≥ 13 | 1.17, CI 0.97-1.41 | **1.71, CI 1.21-2.38** | 1.11, CI 0.89-1.38 | 1.16, CI 0.94-1.43 |
| **Bristol stool scale** | Slow colonic transit (only BSS 1-4) | 1.10, CI 0.95-1.26 | **1.36, CI 1.02-1.80** | 1.11, CI 0.94-1.31 | 1.07, CI 0.87-1.31 |
|  | Normal colonic transit (only BSS 3-4) | 0.96, CI 0.81-1.13 | 1.00, CI 0.70-1.38 | 0.85, CI 0.70-1.03 | 1.04, CI 0.82-1.29 |
|  | Fast colonic transit (only BSS 3-7) | 0.89, CI 0.76-1.03 | 0.77, CI 0.55-1.06 | 0.92, CI 0.77-1.09 | 0.87, CI 0.70-1.08 |
|  | Various (BSS 1/2 and 5/6/7) | 1.05, CI 0.91-1.22 | 0.90, CI 0.65-1.23 | 1.11, CI 0.93-1.31 | 1.03, CI 0.83-1.27 |
| **Pregnancy problems** | Yes | **1.33, CI 1.11-1.59** | **2.16, CI 1.56-2.95** | **1.40, CI 1.14-1.71** | **1.38, CI 1.07-1.75** |
|  | Pregnancy diabetes | 1.66, CI 0.43-5.57 | 2.92, CI 0.43-12.43 | 1.15, CI 0.17-4.84 | 0.90, CI 0.05-5.14 |
|  | Hyperthyroidism | 1.39, CI 0.59-3.04 | 2.83, CI 0.91-7.40 | 0.42, CI 0.07-1.46 | 1.00, CI 0.23-3.02 |
|  | Hypothyroidism | 1.41, CI 0.90-2.19 | 1.10, CI 0.47-2.31 | 1.39, CI 0.82-2.27 | **2.03, CI 1.15-3.47** |
|  | High blood pressure | 0.67, CI 0.19-1.85 | 1.80, CI 0.41-5.67 | 0.94, CI 0.27-2.60 | 0.37, CI 0.02-1.82 |
|  | Preeclampsia | NA | NA | NA | NA |
|  | Hyperemesis gravidarum | 1.37, CI 0.92-2.02 | 1.07, CI 0.51-2.07 | 1.43, CI 0.91-2.20 | 1.04, CI 0.57-1.80 |
|  | Depression | 1.62, CI 0.99-2.62 | 1.79, CI 0.79-3.71 | 1.28, CI 0.70-2.24 | 1.16 CI 0.54-2.29 |
|  | Vaginal bleeding | **1.72, CI 1.15-2.55** | **2.54, CI 1.36-4.62** | **1.55, CI 0.97-2.42** | **1.82, CI 1.06-3.06** |
|  | Acid reflux | 1.19, CI 0.84-1.68 | 1.12, CI 0.61-2.00 | 1.37, CI 0.92-2.01 | 1.12, CI 0.68-1.79 |
|  | Symphysis pubis dysfunction | 0.96, CI 0.65-1.40 | 1.71, CI 0.94-3.03 | 1.06 CI 0.70-1.64 | 0.78, CI 0.44-1.33 |
|  | Other | 0.84, CI 0.60-1.18 | 0.59, CI 0.31-1.07 | 0.98, CI 0.67-1.44 | 0.94, CI 0.60-1.50 |
| **Vomiting in the last 24 hours** | Yes | 1.04, CI 0.83-1.28 | 1.34, CI 0.88-1.98 | 1.01, CI 0.78-1.29 | 0.79, CI 0.56-1.09 |
| **Nausea in the last 24 hours** | Less than 6 hours | 0.91, CI 0.80-1.04 | 1.06, CI 0.83-1.43 | 0.85, CI 0.73-1.00 | 0.92, CI 0.76-1.11 |
|  | More than 6 hours | 0.98, CI 0.82-1.18 | 1.09, CI 0.74-1.56 | 0.98, CI 0.79-1.21 | 0.87, CI 0.66-1.13 |
|  | None | 1.13, CI 0.98-1.30 | 0.85, CI 0.62-1.15 | *1.21, CI 1.03-1.42* | 1.19, CI 0.98-1.45 |
| **General health (self-estimate)** | Very good | **0.87, CI 0.76-0.99** | **0.55, CI 0.40-0.74** | 0.87, CI 0.74-1.01 | 1.05, CI 0.87-1.26 |
|  | Pretty good | 1.05, CI 0.92-1.19 | 1.18, CI 0.90-1.55 | 1.09, CI 0.94-1.27 | 1.02, CI 0.85-1.23 |
|  | Neither good nor bad | **1.39, CI 1.09-1.76** | **2.58, CI 1.72-3.76** | 1.20, CI 0.89-1.60 | 0.88, CI 0.58-1.29 |
|  | Pretty bad | 0.92, CI 0.50-1.57 | 1.60, CI 0.55-3.65 | 0.88, CI 0.42-1.65 | **0.27, CI 0.04-0.87** |
|  | Very bad | 1.47, CI 0.21-6.81 | NA | 2.12, CI 0.30-9.86 | 1.65, CI 0.09-10.25 |
| **Alcohol while pregnant** | Yes | **0.63, CI 0.46-0.84** | **0.43, CI 0.18-0.86** | **0.72, CI 0.51-1.00** | **0.26, CI 0.13-0.47** |
| **Medication during pregnancy** | Yes | **1.43, CI 1.25-1.63** | **2.15, CI 1.63-2.83** | **1.38, CI 1.18-1.61** | **1.34, CI 1.11-1.61** |
| **Drug use during pregnancy** | Asthma and allergy medication | **1.32, CI 1.05-1.65** | **1.80, CI 1.18-2.66** | 1.29, CI 0.98-1.66 | 1.10, CI 0.78-1.52 |
|  | Anxiety, antidepressants, and sleep medication | 1.18, CI 0.94-1.48 | 1.23, CI 0.75-1.91 | 1.19, CI 0.90-1.54 | 1.19, CI 0.86-1.63 |
|  | Prescription free pain medication | 0.98, CI 0.78-1.22 | 1.39, CI 0.91-2.04 | 0.98, CI 0.775-1.25 | **0.68, CI 0.47-0.96** |
|  | Opioids and strong pain medication | **4.09, CI 1.64-10.31** | **6.37, CI 1.41-21.51** | **4.13, CI 1.47-11.12** | **4.60, CI 1.41-13.38** |
|  | Thyroid medication | **1.91, CI 1.54-2.35** | **2.20, CI 1.46-3.22** | **1.78, CI 1.38-2.26** | **2.22, CI 1.68-2.90** |
|  | Blood pressure medication | 1.68, CI 0.79-3.36 | **4.83, CI 1.77-11.20** | 1.99, CI 0.87-4.15 | 1.37, CI 0.40-3.57 |
|  | Stomach acid medication | 1.23, CI 0.91-1.64 | 1.65, CI 0.93-2.71 | 1.33, CI 0.95-1.83 | 0.95, CI 0.59-1.47 |
|  | Other | **1.49, CI 1.22-1.80** | **2.22, CI 1.56-3.11** | **1.40, CI 1.10-1.75** | **1.43, CI 1.08-1.87** |

RPL, Recurrent pregnancy loss; ART, Artificial reproductive technologies; EPDS, Edinburgh Postnatal Depression Scale
