# Supplementary material for: Pre-pregnancy complications - associated factors and wellbeing in early pregnancy: a Swedish cohort study
Source: BMC Pregnancy Childbirth. 2023 Mar 8;23:153. doi: 10.1186/s12884-023-05479-8 (PMC9993650; doi:10.1186/s12884-023-05479-8)
Supplement: Supplementary file 2 — Supplementary material 2 [file 12884_2023_5479_MOESM2_ESM.docx]

**(Web-based questionnaire)**

**MaMi**

The Swedish Maternal Microbiome Project:

**What year were you born (yyyy)?** _________

**How tall are you (cm)?** _________

**What was your weight before you got pregnant (kg)?** _________

**What country were you born in?**

 Sweden

 Outside of Sweden (please state which country): ____________________

**Family**

 Partner (married, cohabitation or living-apart)

 Single

**Do you already have children?**

- No
- Yes, biological child/children (number of children _____)
- Yes, adopted child/children and/or stepchild/stepchildren (number of children _____)

**How many children do you have that attend pre-school/kindergarten** (number of children)? _____

**What is your highest level of completed education?**

 Primary school Senior high school / Upper secondary school

 University/collage Other

**What was your working situation like before you got pregnant?**

- Worked full-time
- Worked part-time
- Long-term sick listed (more than 3 months)
- Unemployed/looking for employment
- Student
- Other: _________

**Are you in contact with animals during your work or spare time?**

  No yes

 **If yes, please state which animal/animals:** dog, cat, rabbit, rodent, reptile, horse, cow, sheep, pig, chicken, other: ____

**Have you been abroad the last 3 months?** Yes/ no

**If so, which country/countries have you been to the last 3 months? _____**

**What pregnancy-week are you currently in? ___________**

**When is you estimated date of delivery?** ________

**What is your weight today (kg)?** _________

**Are you expecting more than one child (twins, triplets etc.)?** Yes/no/don’t know

**For how long have you tried to get pregnant (months)? _________**

**How did you become pregnant? Please tick all applicable alternatives for conception:**

- Natural conception (coitus)
- Medical treatment / ovulation stimulation/induction (e.g. hormone injections, Clomiphene, Metformin)
- Surgical treatment needed (e.g. surgery for endometriosis)
- Assisted reproduction: insemination
- Assisted reproduction: in vitro fertilization (IVF)
- Assisted reproduction: intracytoplasmic sperm injection (ICSI)
- Fertility treatment of the father

**Is this your first pregnancy?** Yes/no

**If no:**

How many times have you been pregnant before (excluding the current pregnancy)? ________

How many children have you given birth to? ________

How many early miscarriages have you had (before 16 weeks)? ________

Has any child you were expecting died during the pregnancy after week 16 (late miscarriage or intrauterine foetal death)? ________

**Comment: _________**

**How many sexual partners have you had intercourse with during the last year?**

- None
- 1
- 2-10
- 11-20
- More than 20
- Prefer not to answer

**Questions about your menstruation (when not on hormonal contraceptives/intrauterine device/pregnant):**

If you have pains during menstruation, how discomforting are your pains on a scale from 0-10 (0 = no pain and 10= severe, disabling pain)? _____

Do you use analgesic/pain killers during your menstruation? Yes/no

Do you experience pain linked to/during ovulation? Yes/no

Do you have regular menstruation? Yes (approx. 28 days), no (≥35 days), no (less than 6 times/year), no (less than 3 times /year)

How many days is your average menstrual cycle, from the first day of one period to the first day of the next? State in days: ___

Do your menstruations usually start with smaller bleeding for over two days before the proper bleeding starts? Yes/no (If yes, small bleeding for how many days: ___)

**Supplementary questions:**

Have you had the diagnosis endometriosis? Yes, diagnosis without surgery/ yes, diagnosis following surgery/no/don’t’ know.

Have you at any time been diagnosed with PCOS (Polycystic Ovary Syndrome)? Yes/no/don’t know.

Have you had acne as an adult? No/ yes, no treatment / yes, local treatment/ yes, antibiotic treatment.


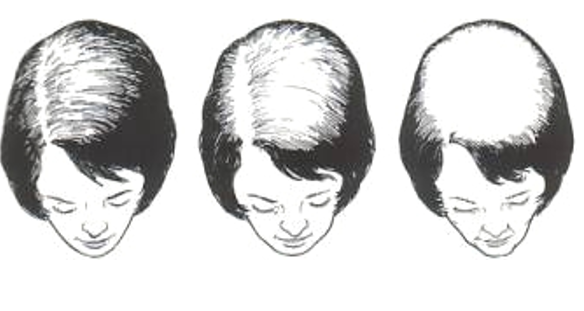
Do you experience problems with thin hair on the top of your head (see picture)?

- No problem with thin hair
- Type 1 according to Ludwig Scale
- Type 2 according to Ludwig Scale
- Type 3 according to Ludwig Scale

Type 1 Type 2 Type 3

Do you experience disturbing body hair? Yes/ no

If yes, please sum up the points according to the pictures below: Score. Total points: _______ (between 0-36 points)

**Questions about screening for cervical cancer (pap smear test or cell specimens): (yes/no/don’t know)**

- Have you ever participated in testing of gynaecological pap smear**:** (for screening of cervical cancer)?
- Have you regularly gone for testing of gynaecological pap smear when summoned?
- Have you at any time had an abnormal HPV-test (Human Papilloma Virus)?
- Have you at any time had cell changes at the gynaecological pap smear?
- Have you at any time undergone biopsy (tissue sampling) from the cervix?
- Have you previously undergone a loop electrical excision procedure (removing a small part of the cervix as treatment of cell changes)?
- Are you vaccinated against HPV (t. ex Gardasil/Silgard/Cervarix)

**Have you had any gynaecological infection in the last 3 months?** Yes/no/don’t know.

**If yes, which infection/infections have you had?**

- Chlamydia
- Gonorrhoea
- Bacterial vaginosis
- Genital herpes
- *Mycoplasma genitalium*
- Vaginal yeast infection/vaginal thrush
- Condyloma
- Trichomoniasis (*Trichomonas vaginalis)*
- Other. If other, which other gynaecological infection have you had in the last 3 months? _____

**Have you used any contraceptives the last year before you got pregnant?** Yes/no

**If yes, which contraceptive/contraceptives have you used in the last year?**

- Birth control pills (including mini-pills)
- Birth control shot (hormone injection every 3 months)
- Birth control implant
- Vaginal hormonal ring
- Copper IUD = Intrauterine device/ coil
- Hormonal IUD = Intrauterine device/ coil
- Condom
- Pessary /diaphragm
- Natural family planning (t. ex: safe periods)
- Other: _______

**How do you rate your general state of health?**

 Very good

 Good

 Neither good or bad

 Poor

 Very poor

**Have you at any time used antibiotics during the last three months BEFORE you got pregnant?** Yes/no

**If yes, for what reason/reasons have you used antibiotics in the 3 months BEFORE pregnancy?**

- Respiratory tract infection (pneumonia, sinusitis)
- Tonsillitis
- Otitis
- Urinary tract infection
- Skin and soft tissue infection (skin, muscles, sore)
- Intestinal infection (bacteria, e.g. salmonella or campylobacter, parasites or traveller’s diarrhoea)
- Abdominal infection (e.g. appendicitis, cholecystitis, diverticulitis)
- Gynaecological infection (e.g. chlamydia, gonorrhoea, bacterial vaginosis)
- Other: ____

**Have you used antibiotics at any time DURING this pregnancy?** Yes/no

**If yes, at what week/weeks of pregnancy: ______**

**If yes, for what reason/reasons have you used antibiotics DURING this pregnancy?**

- Respiratory tract infection (pneumonia, sinusitis)
- Tonsillitis
- Otitis
- Urinary tract infection
- Skin and soft tissue infection (skin, muscles, sore)
- Intestinal infection (bacteria, e.g. salmonella or campylobacter, parasites or traveller’s diarrhoea)
- Abdominal infection (e.g. appendicitis, cholecystitis, diverticulitis)
- Gynaecological infection (e.g. chlamydia, gonorrhoea, bacterial vaginosis)
- Other: ____

**Have you been to the dentist/ dental hygienist DURING the last 3 months before you got pregnant?** Yes/no.

If yes, for what reason/reasons have you been to the dentist/dental hygienist DURING the last 3 months before you got pregnant? _____

**Have you been to the dentist/dental hygienist so far DURING this pregnancy?** Yes/no.

If yes, for what reason/reasons have you been to the dentist/dental hygienist DURING this pregnancy? _____

**Do you smoke/ have you smoked?**

 No, I have never smoked.

 Yes, I did smoke earlier.

 Yes, I smoke now.

 Other smoking habits

If you used to smoke, what year did you stop? _____________

If you smoke, how many cigarettes do you now smoke a day? <1, 1-5, 6-10, >10

What other habits of smoking do you have? _____________

**Do you use “snus” /have you used “snus”?**

 No, I have never used snus.

 Yes, did use snus earlier.

 Yes, use snus now.

 Other habits of using snus

If you used to use snus, what year did you stop? _____________

How many snus do you now take each day? <1, 1-5, 6-10, >10

What other habits of snus do you have? _____________

**How often did you have a drink containing alcoholic during the last three months BEFORE you got pregnant?**

- Never
- Monthly or less
- 2 to 4 times a month
- 2 to 3 times a week
- 4 or more times a week

**How many drinks containing alcohol did you have a typical day when you were drinking during the last three months BEFORE you got pregnant?**

- 1 or 2
- 3 or 4
- 5 or 6
- 7, 8 or 9
- 10 or more

**How often did you have a drink containing alcoholic DURING this pregnancy?**

- Never
- Monthly or less
- 2 to 4 times a month
- 2 to 3 times a week
- 4 or more times a week

**How many drinks containing alcohol do you have a typical day when you are drinking DURING this pregnancy?**

- 1 or 2
- 3 or 4
- 5 or 6
- 7, 8 or 9
- 10 or more

**Have you received treatment for any of the following diseases (prior to pregnancy)?** Yes/no/don’t know

- Diabetes
  - Type 1
  - Type 2
- Thyroid disease
  - Overactive thyroid (hyperthyroidism)
  - Underactive thyroid (hypothyroidism, treated with e.g. Levaxin)
- High blood pressure (hypertension)
- Cancer treated with chemotherapy, radiotherapy of hormone therapy.
- Multiple sclerosis (MS)
- Rheumatism or other autoimmune diseases (e.g. SLE or scleroderma)
- Crohn’s disease
- Ulcerative colitis
- Allergy
  - Asthma
  - Eczema
  - Hay fever/Allergic rhinitis
  - Other allergy
- Other disease, please explain which disease: _______

**Has a doctor or other healthcare professional ever told you that you might have an eating disorder?** Yes/no/don’t know

**If yes, when a doctor or other healthcare professional told you that you might have an eating disorder, which eating disorder did they say you have (please mark all relevant):**

- 1. Anorexia
  2. Bulimia
  3. Binge eating disorder
  4. Other specified or unspecified eating disorder
  5. Other eating disorder __________

**Have any of your biological parents, siblings or children ever had an eating disorder?**

Yes/no/don’t know

**So far during this pregnancy, do you have or have you had any complications/ medical problems?**

**What pregnancy-related complications have you experienced during this pregnancy?** (Yes/no/don’t know)

- Gestational diabetes
- Thyroid disease
  - Overactive thyroid (hyperthyroidism)
  - Underactive thyroid (hypothyroidism, treated with e.g. Levaxin)
- High blood pressure (hypertension) (that you did not have before pregnancy)
- Pre-eclampsia (toxaemia)
- Hyperemesis gravidarum (severe morning sickness*)*
- Depression
- Vaginal bleeding/threatened abortion
- Heartburn/dyspepsia
- Symphysis pubis dysfunction/ pelvic girdle pain
- Other

If other, please describe which*_____*

**Did you take any medications regularly before you got pregnant?** Yes/no

If yes, what medications did you take regularly before you got pregnant?

- Non-prescription/over-the-counter analgesics/painkillers (e.g. Alvedon, Ipren)

- Opioids, stronger analgesics/painkillers (e.g. Morfin, Oxynorm)

- Allergy medications (e.g. antihistamines like Aerius, Loratidin)

- Asthma medications (e.g. Inhalations like Bricanyl, Pulmicort, Ventoline)

- Antidepressant medications (e.g. Citalopram, Sertralin)

- Sedative and antianxiety agents (e.g. Sobril, Stesolid)

- Hypnotics/sleeping pills (e.g. Imovane, Propavan)

- Thyroid hormone (e.g. Levaxin)

- Antihypertensive/blood pressure medications (e.g. Enalapril, Furix, Lasix, Spironolakton, Amlodipin, Felodipin, Atacand, Losartan, Seloken, Metroprolol)

- Medications decreasing the gastric acid production (e.g. Proton-pump-inhibitors like Omeprazol or similar)

- Other

What other medications did you take regularly before you got pregnant? ________________

**Do you take any medications regularly since you got pregnant?** Yes/no

If yes, what medications do you take regularly since you got pregnant?

- Non-prescription/over-the-counter analgesics/painkillers (e.g. Alvedon, Ipren)

- Opioids, stronger analgesics/painkillers (e.g. Morfin, Oxynorm)

- Allergy medications (e.g. antihistamines like Aerius, Loratidin)

- Asthma medications (e.g. Inhalations like Bricanyl, Pulmicort, Ventoline)

- Antidepressant medications (e.g. Citalopram, Sertralin)

- Sedative and antianxiety agents (e.g. Sobril, Stesolid)

- Hypnotics/sleeping pills (e.g. Imovane, Propavan)

- Thyroid hormone (e.g. Levaxin)

- Antihypertensive/blood pressure medications (e.g. Enalapril, Furix, Lasix, Spironolakton, Amlodipin, Felodipin, Atacand, Losartan, Seloken, Metroprolol)

- Medications decreasing the gastric acid production (e.g. Proton-pump-inhibitors like Omeprazol or similar)

- Other

What other medications did you take regularly since you got pregnant? _________________

**In the last 24 hours, for how long have you felt nauseated or sick to your stomach?**

- Not at all
- 1 hour or less
- 2-3 hours
- 4-6 hours
- More than 6 hours

**In the last 24 hours have you vomited or thrown up?**

- 7 times or more
- 5-6 times
- 3-4 times
- 1-2 times
- I did not throw up

**In the last 24 hours how many times have you had retching or dry heaves without bringing anything up?**

- No time
- 1-2 times
- 3-4 times
- 5-6 times
- 7 more times

**Being pregnant and expecting a child can be intense and much can occur. We hope that you and the child you are expecting are well. If something has gone wrong during this pregnancy we would be grateful if you could tell us what has happened: _____________________________________________________________**

**How do you rate your general state of health?**

 Very good; Good; Neither good or bad; Poor; Very poor

**What pregnancy-week are you currently in?**

**When is you estimated date of delivery?** ________

**What is your weight today (kg)?** _________

**Are you expecting more than one child (twins, triplets etc.)?** Yes/no/don’t know

Comment: _________

**Have you used antibiotics at any time during this pregnancy?** Yes/no

**If yes, at what week/weeks of pregnancy: ______**

**If yes, for what reason/reasons have you used antibiotics during this pregnancy?**

- Respiratory tract infection (pneumonia, sinusitis)
- Tonsillitis
- Otitis
- Urinary tract infection
- Skin and soft tissue infection (skin, muscles, sore)
- Intestinal infection (bacteria, e.g. salmonella or campylobacter, parasites or traveller’s diarrhoea)
- Abdominal infection (e.g. appendicitis, cholecystitis, diverticulitis)
- Gynaecological infection (e.g. chlamydia, gonorrhoea, bacterial vaginosis)
- Other: ____

**Have you been to the dentist/dental hygienist so far during this pregnancy?** Yes/no

**If yes, for what reason/reasons have you been to the dentist/dental hygienist during this pregnancy? _____**

**Do you smoke/ use snus now?**

If you smoke, how many cigarettes do you smoke a day? <1, 1-5, 6-10, >10

If you use snus, how many snus do you take a day? <1, 1-5, 6-10, >10

**How often did you have a drink containing alcoholic DURING this pregnancy?**

- Never
- Monthly or less
- 2 to 4 times a month
- 2 to 3 times a week
- 4 or more times a week

**How many drinks containing alcohol do you have a typical day when you are drinking DURING this pregnancy?**

- 1 or 2
- 3 or 4
- 5 or 6
- 7, 8 or 9
- 10 or more

**So far during this pregnancy, do you have or have you had any complications/medical problems?**

**What pregnancy-related complications have you experienced during this pregnancy?** (Yes/no/don’t know)

- Gestational diabetes
- Thyroid disease
  - Overactive thyroid (hyperthyroidism)
  - Underactive thyroid (hypothyroidism, treated with e.g. Levaxin)
- High blood pressure (hypertension) (that you did not have before pregnancy)
- Pre-eclampsia (toxaemia)
- Hyperemesis gravidarum (severe morning sickness*)*
- Depression
- Vaginal bleeding/threatened abortion
- Heartburn/dyspepsia
- Symphysis pubis dysfunction/pelvic girdle pain
- Other

If other, please describe which: *_____*

**Do you take any medications regularly during this pregnancy?** Yes/no

If yes, what medications do you take regularly during this pregnancy?

- Non-prescription/over-the-counter analgesics/painkillers (e.g. Alvedon, Ipren)

- Opioids, stronger analgesics/painkillers (e.g. Morfin, Oxynorm)

- Allergy medications (e.g. antihistamines like Aerius, Loratidin)

- Asthma medications (e.g. Inhalations like Bricanyl, Pulmicort, Ventoline)

- Antidepressant medications (e.g. Citalopram, Sertralin)

- Sedative and antianxiety agents (e.g. Sobril, Stesolid)

- Hypnotics/sleeping pills (e.g. Imovane, Propavan)

- Thyroid hormone (e.g. Levaxin)

- Antihypertensive/blood pressure medications (e.g. Enalapril, Furix, Lasix, Spironolakton, Amlodipin, Felodipin, Atacand, Losartan, Seloken, Metroprolol)

- Medications decreasing the gastric acid production (e.g. Proton-pump-inhibitors like Omeprazol or similar)

- Other

What other medications do you take regularly since you got pregnant? _________________

**In the last 24 hours, for how long have you felt nauseated or sick to your stomach?**

- Not at all
- 1 hour or less
- 2-3 hours
- 4-6 hours
- More than 6 hours

**In the last 24 hours have you vomited or thrown up?**

- 7 times or more
- 5-6 times
- 3-4 times
- 1-2 times
- I did not throw up

**In the last 24 hours how many times have you had retching or dry heaves without bringing anything up?**

- No time
- 1-2 times
- 3-4 times
- 5-6 times
- 7 more times

**Do you take iron supplements?** Yes/no

**Do you take other vitamin-, mineral or dietary supplements?** Yes/no

**What vitamin-, mineral- or dietary supplements do you take? _______

Have you during the last days consumed products containing probiotics (e.g. Proviva, Actimel) or dietary supplements (e.g. ProBi-mage or similar)?**

 No Yes, (please state which one of you know the name)_____________________

**Are you vegetarian?** Yes/no

**Are you vegan?** Yes/no

**Do you eat fish?** Yes/no

**How often do you drink/eat one of the following?
Think about the last month.**

**Daily Several Once More rarely times a week than once a a week week**

**Sweet drinks with sugar
(e.g. soda, juice)**

**Sweet drinks without sugar**

**(e.g. diet soda)**

**Multigrain bread**

**Fruit**

**Vegetables /Root vegetables**

**Have you in the last month experienced discomfort from:** (Yes/no)

Diarrhoea?

Constipation?

Bloating/gases?

Other discomfort from stomach/intestines?

If yes, please describe: _______________________


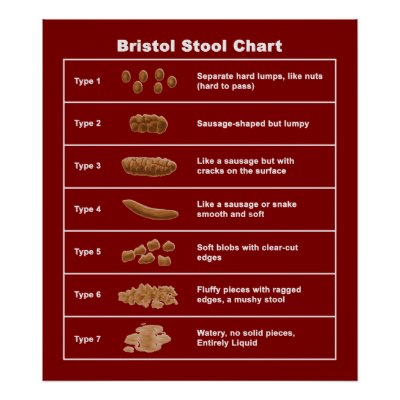
**What did your stool look like during the past two weeks? (see picture)**

Tick all the boxes you have had. You can pick more than one alternative.

 Type 1. Separate hard lumps, like nuts (hard to pass)

 Type 2. Sausage shaped but lumpy.

 Type 3. Like a sausage but with cracks in the surface

 Type 4. Like a sausage or snake, smooth and soft.

 Type 5. Soft blobs with clear-cut edges (easy to pass)

 Type 6. Fluffy pieces with ragged edges, a mushy stool.

 Type 7. Watery, no solid pieces, entirely liquid (diarrhoea).

The following questions ask about your feelings and thoughts during **the past month**. In each question, you will be asked how often you felt or thought a certain way.

**In the last month, how often have you been/felt:**

|  | Never | Almost  Never | Some-  Times | Fairly  Often | Very  Often |
| --- | --- | --- | --- | --- | --- |
| a) Unable to control the important things in your life? | 0 | 1 | 2 | 3 | 4 |
| b) Confident about your ability to handle your personal problems? | 0 | 1 | 2 | 3 | 4 |
| c) Things were going your way? | 0 | 1 | 2 | 3 | 4 |
| d) Difficulties were piling up so high that you could not overcome them? | 0 | 1 | 2 | 3 | 4 |

**Please check the answer that comes closest to how you have felt in the past 7 days, not just how you feel today**

a) I have been able to laugh and see As much as I always could

the funny side of things not quite so much now

 Definitely not so much now

 Not at all

b) I have looked forward with enjoyment to things As much as I ever did

 Rather less than I used to

 Definitely less than I used to

 Hardly at all

c) I have blamed myself unnecessarily Yes, most of the time

when things went wrong Yes, some of the time

 Not very often

 No, never

d) I have been anxious or worried No, not at all

for no good reason Hardly ever

 Yes, sometimes

 Yes, very often

e) I have felt scared or panicky Yes, quite a lot

for no very good reason Yes, sometimes

 No, not much

 No, not at all

f) Things have been getting
 on top of me

 Yes, most of the time I haven’t been able to cope at all

 Yes sometimes I haven’t been coping as well as usual No, most of the time I have coped quite well No, I have been coping as well as ever

g) I have been so unhappy Yes, most of the time
that I have had difficulty sleeping Yes, sometimes
 Not very often
 No, not at all

h) I have felt sad or miserable
 Yes, most of the time
 Yes, quite often
 Not very often
 No, not at all

i) I have been so unhappy Yes, most of the time
that I have been crying Yes, quite often
 Only occasionally
 No, never

j) The thought of harming myself Yes, quite often
has occurred to me Sometimes
 Hardly ever
 Never
